# Cooperation of long noncoding RNA LOC100909675 and transcriptional regulator CTCF modulates *Cdk1* transcript to control astrocyte proliferation

**DOI:** 10.1016/j.jbc.2023.105153

**Published:** 2023-08-09

**Authors:** Ronghua Wu, Haixu Lin, Wei Zhang, Ying Sun, Xiaowei Qian, Ge Lin, Chao Ma, Zhangji Dong, Bin Yu, Liu Yang, Yan Liu, Mei Liu

**Affiliations:** 1 Key Laboratory of Neuroregeneration of Jiangsu and Ministry of Education, Co-innovation Center of Neuroregeneration, NMPA Key Laboratory for Research and Evaluation of Tissue Engineering Technology Products, Nantong University, Nantong, China; 2 Departement of Neurosurgery, Affiliated Hospital of Nantong University, Nantong, China

**Keywords:** lncRNA LOC100909675, astrocyte, Cdk1 (cyclin dependent kinase 1), CTCF (CCCTC-binding factor), spinal cord injury

## Abstract

Astrocyte activation and proliferation contribute to glial scar formation during spinal cord injury (SCI), which limits nerve regeneration. The long noncoding RNAs (lncRNAs) are involved in astrocyte proliferation and act as novel epigenetic regulators. Here, we found that lncRNA-LOC100909675 (LOC9675) expression promptly increased after SCI and that reducing its expression decreased the proliferation and migration of the cultured spinal astrocytes. Depletion of LOC9675 reduced astrocyte proliferation and facilitated axonal regrowth after SCI. LOC9675 mainly localized in astrocytic nuclei. We used RNA-seq to analyze gene expression profile alterations in LOC9675-depleted astrocytes and identified the cyclin-dependent kinase 1 (*Cdk1*) gene as a hub candidate. Our RNA pull-down and RNA immunoprecipitation assays showed that LOC9675 directly interacted with the transcriptional regulator CCCTC-binding factor (CTCF). Dual-luciferase reporter and chromatin immunoprecipitation assays, together with downregulated/upregulated expression investigation, revealed that CTCF is a novel regulator of the Cdk1 gene. Interestingly, we found that with the simultaneous overexpression of CTCF and LOC9675 in astrocytes, the *Cdk1* transcript was restored to the normal level. We then designed the deletion construct of LOC9675 by removing its interacting region with CTCF and found this effect disappeared. A transcription inhibition assay using actinomycin D revealed that LOC9675 could stabilize *Cdk1* mRNA, while LOC9675 depletion or binding with CTCF reduced *Cdk1* mRNA stability. These data suggest that the cooperation between CTCF and LOC9675 regulates *Cdk1* transcription at a steady level, thereby strictly controlling astrocyte proliferation. This study provides a novel perspective on the regulation of the *Cdk1* gene transcript by lncRNA LOC9675.

Spinal cord injury (SCI) remains a clinical challenge among traumatic diseases of the central nervous system (CNS) and often results in severe functional loss of injured nerves, and there are only a few effective therapeutic solutions for SCI owing to its complex dysfunction etiology involving multiple systems. Failed regeneration after SCI is due to limitations in intrinsic regenerative ability, inhibitory factors in the injured microenvironment, and glial scar formation (1). Astrocytes are the most abundant glial cells in the CNS and perform various functions, including neurogenesis, neuronal survival, neurotransmission, and immune surveillance (2, 3, 4). In addition to maintaining normal neuronal functions in the CNS, when suffering trauma, inflammation, or other pathological changes, astrocytes exhibit morphological changes, rapidly increasing their proliferation and migration, which is also called astrocytic activation. During SCI, activated astrocytes generate a large amount of extracellular matrix, which is the main component of the glial scar. Although activated astrocytes initially limit the spread of damage, glial scars eventually hinder axon regeneration (4, 5). Astrocytes exhibit various features during the different phases of spinal cord damage, indicating that astrocytes are regulated by multiple molecules.

Long noncoding RNAs (lncRNAs) belong to the noncoding RNA family and have a transcript length of >200 nucleotides (6, 7). Recent studies have shown promising progress in the regulation of gene expression using lncRNAs. Thus far, our understanding of lncRNA regulation encompasses multiple levels, including its interactions with DNA, RNA, and proteins (8, 9, 10, 11, 12). Gene expression profile analyses have revealed significant changes in lncRNA expression in rodent SCI (10, 13). The lncRNAs contribute to the expression of a variety of mRNAs, participating in neuronal survival, glial activation, and other processes, and are considered new regulators of the pathological process after SCI (14, 15).

A recent publication reported the expression profiles of lncRNAs after rat SCI (16). We studied one lncRNA, named LOC100909675 (LOC9675), whose transcript showed rapid upregulation and was maintained at a higher level after SCI. LOC9675 localizes at Chr.4q11 (Genbank accession number: NR_110709) of the rat genome, and full length of 1270 nt was obtained using the 5′- and 3′—rapid amplification of complementary DNA (cDNA) ends (RACE) technique. Herein, we reported the effects of LOC9675 depletion in astrocytes *in vitro* and *in vivo* and further studied its molecular mechanism.

## Results

### Effects of LOC9675 depletion on axonal length, astrocyte proliferation, and migration

**Figure 1:**
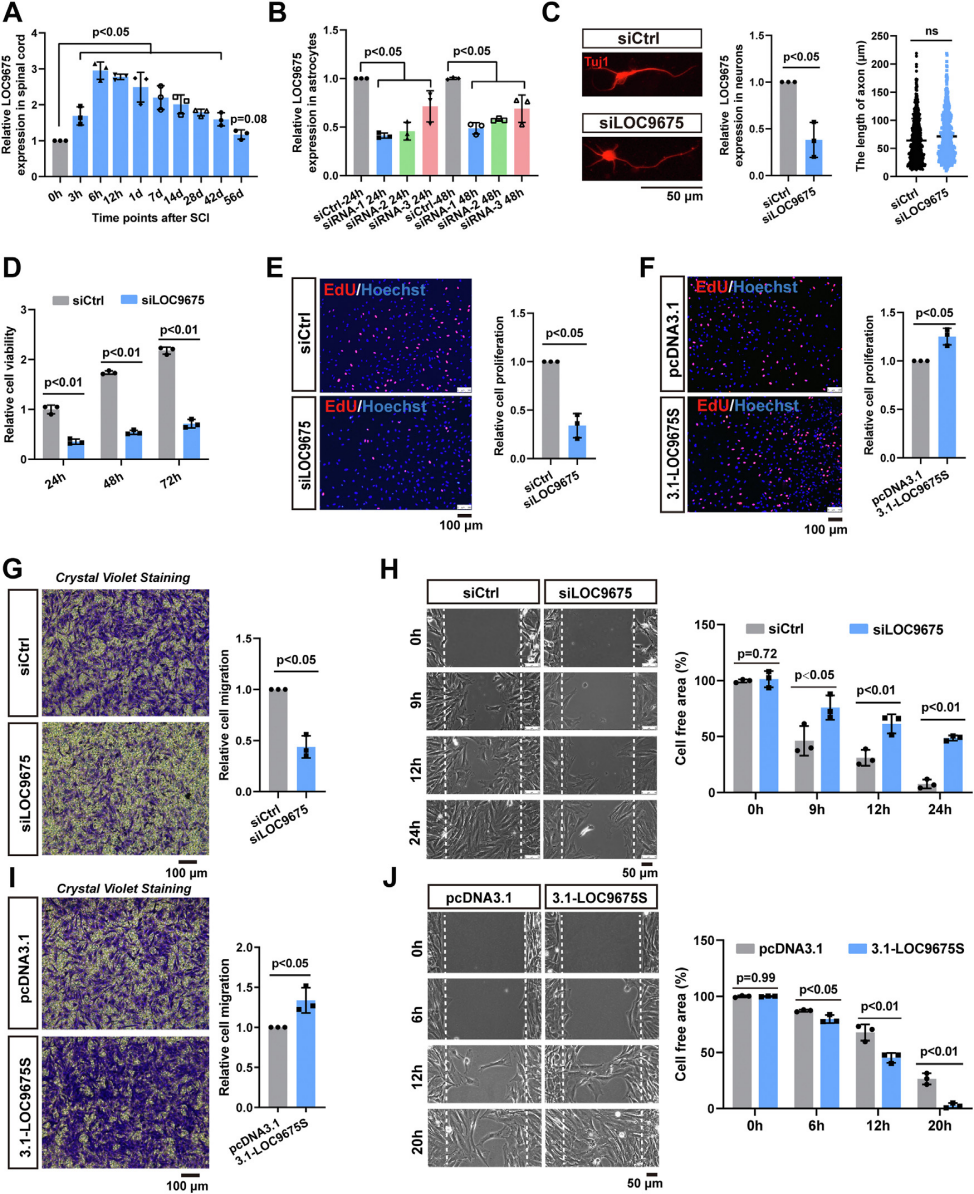
**Effects of LOC9675 knockdown on the axonal length, astrocytic proliferation, and migration.***A*, qRT-PCR results of LOC9675 expression after spinal cord hemisection injury in rats. LOC9675 expression at 0 h was normalized as 1, and (3 h) = 1.69 ± 0.25, (6 h) = 2.95 ± 0.24, (12 h) = 2.78 ± 0.07, (1 day) = 2.49 ± 0.41, (7 days) = 2.20 ± 0.33, (14 days) = 2.01 ± 0.26, (28 days) = 1.79 ± 0.09, (42 days) = 1.59 ± 0.18, (56 days) =1.17 ± 0.13. The data are shown as the mean ± SD. Data were analyzed using one-way ANOVA followed by Tukey’s post hoc test (*p* < 0.05). Unpaired Student's *t* test was used to compare two groups (*versus* 0 h, n = 3). The *p* values are shown on the panel. *B*, graph showing the knockdown efficiency of LOC9675 siRNA in astrocytes. After siRNA-1 treatment for 24 and 48 h, relative levels of LOC9675 expression decreased by 59% and 51.4%, respectively. After siRNA-2 treatment for 24 and 48 h, relative levels of LOC9675 expression decreased by 54.3% and 42.6%, respectively. After siRNA-3 treatment for 24 and 48 h, relative levels of LOC9675 expression decreased by 28.7% and 31.2%, respectively. The mRNA expression level of the control siRNA was normalized to 1. The data are shown as the mean ± SD. Data were analyzed using one-way ANOVA followed by Tukey’s post hoc test (*p* < 0.05). Unpaired Student's *t* test was used to compare two groups (*versus* siCtrl, n = 3). The *p* values are shown on the panel. *C*, effect of siLOC9675 treatment on neuronal axonal growth. The *left panel* shows representative IF images of samples stained with Tuj1 antibody (a marker for neurons), the scale bar represents 50 μm. The *middle panel* shows the knockdown efficiency of LOC9675 siRNA in neurons. The *right panel* shows the statistical graph of axonal length, the axonal length of siCtrl = 62.58 ± 3.462 μm; siLOC9675 = 70.97 ± 2.511 μm, n = 600 neurons. The data are shown as the mean ± SD. Data were analyzed using unpaired Student’s *t* test (*versus* siCtrl, n = 3). The *p* values are shown on the panel. *D*, cell Counting Kit-8 (CCK-8) assay showing that astrocytic viability decreased by 64.6%, 68.9%, and 67.3% after LOC9675 siRNA treatment for 24, 48, and 72 h, respectively. The data are shown as the mean ± SD. Data were analyzed using two-way ANOVA followed by Bonferroni's post hoc test (*p* < 0.05). Unpaired Student's *t* test was used to compare two groups (*versus* siCtrl, n = 3). The *p* values are shown on the panel. *E*, 5-ethynyl-2′-deoxyuridine (EdU) assay showing that astrocytic proliferation decreased by 66% after LOC9675 siRNA treatment. *Left panel*: representative EdU images. *Right panel*: statistical analysis. The data are shown as the mean ± SD. Data were analyzed using unpaired Student’s *t* test (*versus* siCtrl, n = 3). The *p* value is shown on the panel. *F*, EdU results showed that astrocytic proliferation increased by 25.1% following LOC9675 overexpression treatment. The data are shown as the mean ± SD. Data were analyzed using unpaired Student’s *t* test (*versus* siCtrl, n = 3). The *p* value is shown on the panel. *G*, transwell assay showing that astrocyte migration decreased by 56.2% after LOC9675 siRNA. *Left panel*: representative crystal violet staining images. *Right panel*: statistical analysis. The data are shown as the mean ± SD. Data were analyzed using unpaired Student’s *t* test (*versus* siCtrl, n = 3). The *p* value is shown on the panel. *H*, ibidi chamber assay showing the width of the cell-free gap. *Left panel*: representative bright-field image. *Right panel*: statistical analysis. The cell-free area at 0 h was normalized as 100%, siCtrl group: (9 h) = (46.20 ± 13.22)%, (12 h) = (31.00 ± 7.02)%, (24 h) = (7.60 ± 4.01)%; siLOC9675 group: (9 h) = (75.87 ± 10.91)%, (12 h) = (61.33 ± 8.62)%, (24 h) = (48.70 ± 2.23)%. The data are shown as the mean ± SD. Data were analyzed using two-way ANOVA followed by Bonferroni's post hoc test (*p* < 0.05). Unpaired Student's *t* test was used to compare two groups (*versus* siCtrl, n = 3). The *p* values are shown on the panel. *I*, transwell assay showing that astrocyte migration increased by 33.8% after LOC9675 overexpression treatment. *Left panel*: representative crystal *violet* staining images. *Right panel*: statistical analysis. The data are shown as the mean ± SD. Data were analyzed using unpaired Student’s *t* test (*versus* siCtrl, n = 3). The *p* value is shown on the panel. *J*, ibidi chamber assay showing the width of the cell-free gap. *Left panel*: representative bright-field image. *Right panel*: statistical analysis. The cell-free area of 0 h was normalized as 100%, pcDNA3.1 group: (6 h) = (87.19 ± 0.96)%, (12 h) = (67.79 ± 7.24)%, (20 h) = (26.53 ± 4.93)%; 3.1-LOC9675 group: (6 h) = (79.73 ± 3.53)%, (12 h) = (45.20 ± 4.37)%, (20 h) = (2.63 ± 2.25)%. The data are shown as the mean ± SD. Data were analyzed using two-way ANOVA followed by Bonferroni's post hoc test (*p* < 0.05). Unpaired Student's *t* test was used to compare two groups (*versus* pcDNA3.1, n = 3). The *p* values are shown on the panel. IF, immunofluorescence; pcDNA, plasmid cloning DNA; qRT-PCR, quantitative reverse transcription PCR.

We first verified the expression pattern of LOC9675 after rat SCI. The spinal cord hemisection injury model was established as described previously (17). LOC9675 expression was studied in injured spinal cord tissues at 3 h, 6 h, and 12 h, and on days 1, 7, 14, 28, 42, and 56 after SCI by quantitative reverse transcription PCR (qRT-PCR). As shown in Figure 1*A*, the expression of LOC9675 promptly increased and peaked at 6 h. Subsequently, LOC9675 exhibited a slight decrease but was maintained at a higher level than that of the control (0 h), and it almost returned to normal level on the 56th day after SCI.

Considering that neurons and astrocytes are the main cell components in the spinal cord, we investigated the effects of knocking down LOC9675 on primary cultured spinal neurons from E14-days Sprague-Dawley (SD) rats and spinal astrocytes from P1-day SD rats. As shown in Figure 1*B*, LOC9675 siRNA-1 was effective in astrocytes, and we used this siRNA to treat cultured spinal neurons and astrocytes, respectively. After siLOC9675 transfection in neurons for 48 h, the neurons were immunostained with Tuj1, and the results showed no notable changes in axonal length (Fig. 1*C*). We examined spinal astrocytes and found that siLOC9675 treatment substantially inhibited astrocyte viability, proliferation, and migration. Compared to the siCtrl group, depletion of LOC9675 resulted in a 64.6%, 68.9%, and 67.3% decrease in cell viability at 24 h, 48 h, and 72 h, respectively, as detected by the Cell Counting Kit-8 (CCK-8) assay (Fig. 1*D*), and a 66% decrease in cell proliferation as detected by the 5-ethynyl-2′-deoxyuridine (EdU) incorporation assay (Fig. 1*E*). We also tested the effect of LOC9675 overexpression in astrocytes and found that cell proliferation increased by 25.1% (Fig. 1*F*). The results of the Transwell test and wound healing assays revealed that LOC9675 knockdown led to a 56.2% inhibition of cellular vertical migration (Fig. 1*G*) and a 29.7%, 30.3%, and 41.1% decrease in cellular horizontal migration at 9 h, 12 h, and 24 h, respectively (Fig. 1*H*). LOC9675 overexpression in astrocytes led to a 33.8% increase in cellular vertical migration (Fig. 1*I*) and a 7.5%, 22.6%, and 23.9% increase in cellular horizontal migration at 6 h, 12 h, and 20 h, respectively (Fig. 1*J*).

These results suggest that depletion of LOC9675 in astrocytes significantly inhibits proliferation and migration *in vitro*, which may limit glial activation and scar formation *in vivo*. Therefore, we tested whether LOC9675 knockdown improves functional recovery after SCI in rats by restricting astrocyte proliferation and migration.

### Effect of LOC9675 depletion on functional recovery after rat SCI

**Figure 2:**
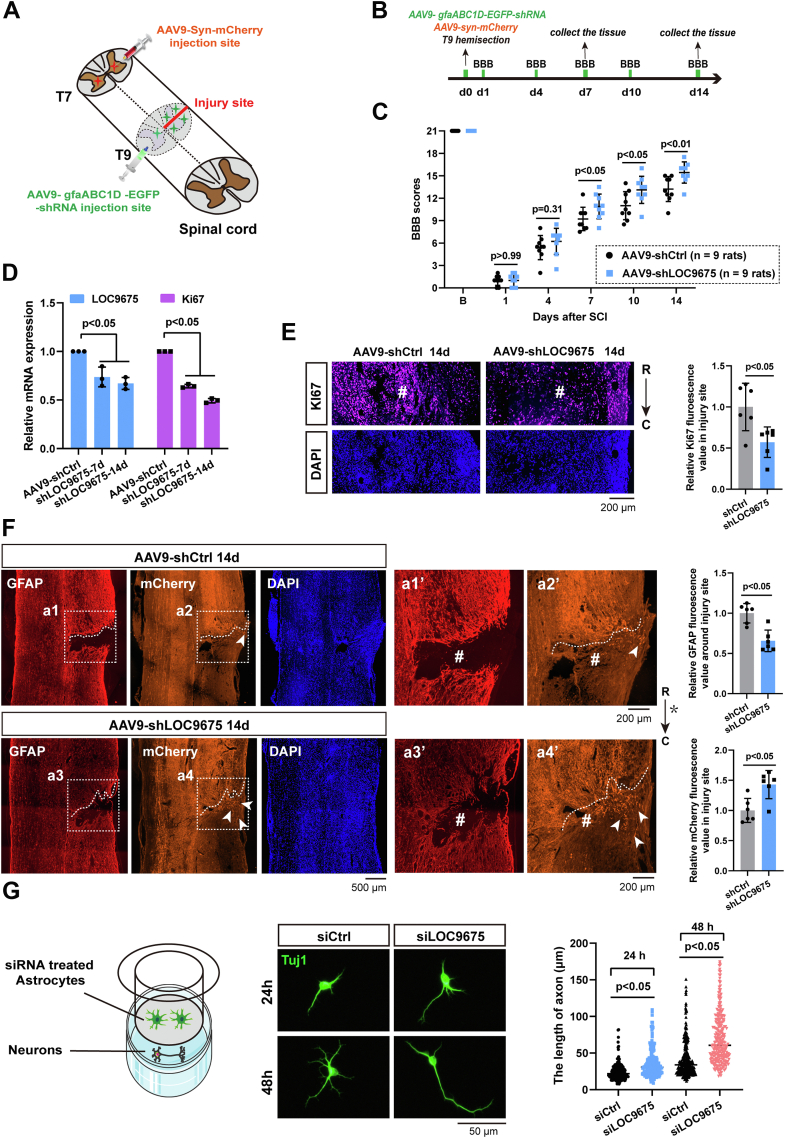
**LOC9675 knockdown promoted functional recovery after spinal cord injury in rats.***A*, diagrammatic sketch of AAV virus injection and T9 hemisection. *B*, experimental timeline of AAV injection and spinal cord hemisection injury in rats. *C*, BBB scores of animals treated with AAV9-shCtrl or AAV9-shLOC9675. “B” strand before the injury. The data are shown as the mean ± SD. Data were analyzed using two-way ANOVA followed by Bonferroni's post hoc test (*p* < 0.05). Unpaired Student's *t* test was used to compare two groups (AAV9-shLOC9675 *versus* AAV9-shCtrl), *p* value is shown on the panel, n = 9 rats for each group. *D*, qRT-PCR results showing the relative expression of LOC9675 and Ki67 after aav9-shLOC9675 treatment at 7 and 14 days, respectively. The data are shown as the mean ± SD. Data were analyzed using one-way ANOVA followed by Tukey’s post hoc test (*p* < 0.05). Unpaired Student's *t* test was used to compare two groups (*versus* AAV9-shCtrl, n = 3). The *p* values are shown on the panel. *E*, *left panel*, representative immunofluorescence (IF) results showing the expression of Ki67 proteins at the injury site (T9, labeled #) 14 days after SCI. *Right panel*: statistical analyses. *R*, rostral; *C*, caudal; the scale bar represents 200 μm. The data are shown as the mean ± SD. Data were analyzed using unpaired Student’s *t* test (*versus* AAV9-shCtrl, n = 6 rats). The *p* value is shown on the panel. *F*, *left panel*, representative immunofluorescence (IF) results showing the localization of GFAP proteins, Syn-mCherry, and DAPI 14 days after SCI. The *dashed line* and the *white triangle* indicate the approximate boundary of the glial scar and a small number of nerve fibers, respectively, the scale bar represents 200 μm. Panels a1′–a4′ are magnifications of the *white squares* in Panels a1–a4, respectively, the scale bar represents 500 μm. *Right panel*, statistical analysis. The injured site (labeled #); the *asterisk* represents the injured side, *R* indicates the rostral side, and *C* indicates the caudal side. The data are shown as the mean ± SD. Data were analyzed using unpaired Student’s *t* test (*versus* AAV9-shCtrl, n = 6 rats). The *p* value is shown on the panel. *G*, *left panel*: schematic of the coculture experiment. *Middle panel*: immunostaining of neurons using Tuj1 antibodies. *Right panel*, statistical results of the axonal length, ((24 h): siCtrl = 24.89 ± 0.89 μm, siLOC9675 = 35.39 ± 1.21 μm; (48 h): siCtrl = 41.48 ± 1.46 μm, siLOC9675 = 71.52 ± 2.37 μm); n = 200 neurons. The data are shown as the mean ± SD. Data were analyzed using unpaired Student’s *t* test (*versus* siCtrl, n = 3). The *p* values are shown on the panel. AAV, adeno-associated virus; BBB, Basso, Beattie, and Bresnahan; DAPI, 4′,6-diamidino-2-phenylindole; GFAP, glial fibrillary acidic protein; qRT-PCR, quantitative reverse transcription; SCI, spinal cord injury.

We investigated the effects of astrocyte-specific LOC9675 depletion on functional recovery after SCI *in vivo*. A schematic of the procedure and time schedule is shown in Figure 2, *A* and *B*. A rat model of spinal cord hemisection was established, and adeno-associated virus (AAV)9-gfaABC1D promoter-EGFP-LOC9675 shRNA (AAV9-shLOC9675) was injected to specifically deplete LOC9675 in activated astrocytes. Control rats were injected with AAV9-gfaABC1D promoter-EGFP-control shRNA (AAV9-shCtrl). As shown in Figure 2*C*, compared to the control, the Basso, Beattie, and Bresnahan (BBB) score of AAV9-shLOC9675 treated rats revealed improved functional recovery, with a significant difference at 14 days after injury. The expression of a marker of proliferation, *Mki67*, significantly decreased the mRNA level at 7 and 14 days after injury following LOC9675 knockdown (Fig. 2*D*), and the immunostaining of Ki67 was also notably reduced at 14 days after injury (Fig. 2*E*, #), suggesting that cell proliferation was inhibited.

We fixed the tissues at 14 days after injury and performed immunostaining using anti-glial fibrillary acidic protein (GFAP) and anti-chondroitin sulfate proteoglycans (CSPG) antibodies. CSPG is a member of a large family of extracellular matrix proteins involved in glial scar formation. After SCI, activated astrocytes secrete CSPGs, and CSPGs constitute a major barrier to nascent axons (18). The results showed that compared to the control group, siLOC9657 treatment led to a significant decrease in GFAP expression (Fig. 2*F*), and the area of CSPG expression showed a decreasing trend; however, the difference was not statistically significant (Fig. S1). The regrowing axons of injured corticospinal neurons were traced using the AAV9-pSyn-mCherry virus. As shown in Figure 2*F*, we found that the mCherry-traced injured axons (labeled with white arrows in Fig. 2*F*a2′) almost stopped at the rostral terminal of the injured site (labeled with # in Fig. 2*F*, a1′ and a2′) in the control. siLOC9657 treatment resulted in a significant increase in several axons (white arrows in Fig. 2*F*a4′) that directly accessed the injured site (labeled # in Fig. 2*F*, a3′ and a4′). These results suggest that depletion of LOC9657 could improve functional recovery and promote axonal regeneration.

Furthermore, we verified whether siLOC9675-treated astrocytes could improve axonal growth *in vitro* using a Transwell chamber. After treatment with siCtrl or siLOC9675 for 24 h, astrocytes were resuspended and plated in the upper chamber, while spinal neurons were cultured in the lower chamber. The neurons were observed by Tuj1 immunostaining after 24 or 48 h of coculture, and we found that compared to the siCtrl group, siLOC9675 treatment showed a 42.2% or 72.4% increase in axonal length, respectively (Fig. 2*G*).

### Cellular localization of LOC9675 in astrocytes and gene expression profile alteration after LOC9675 knockdown

**Figure 3:**
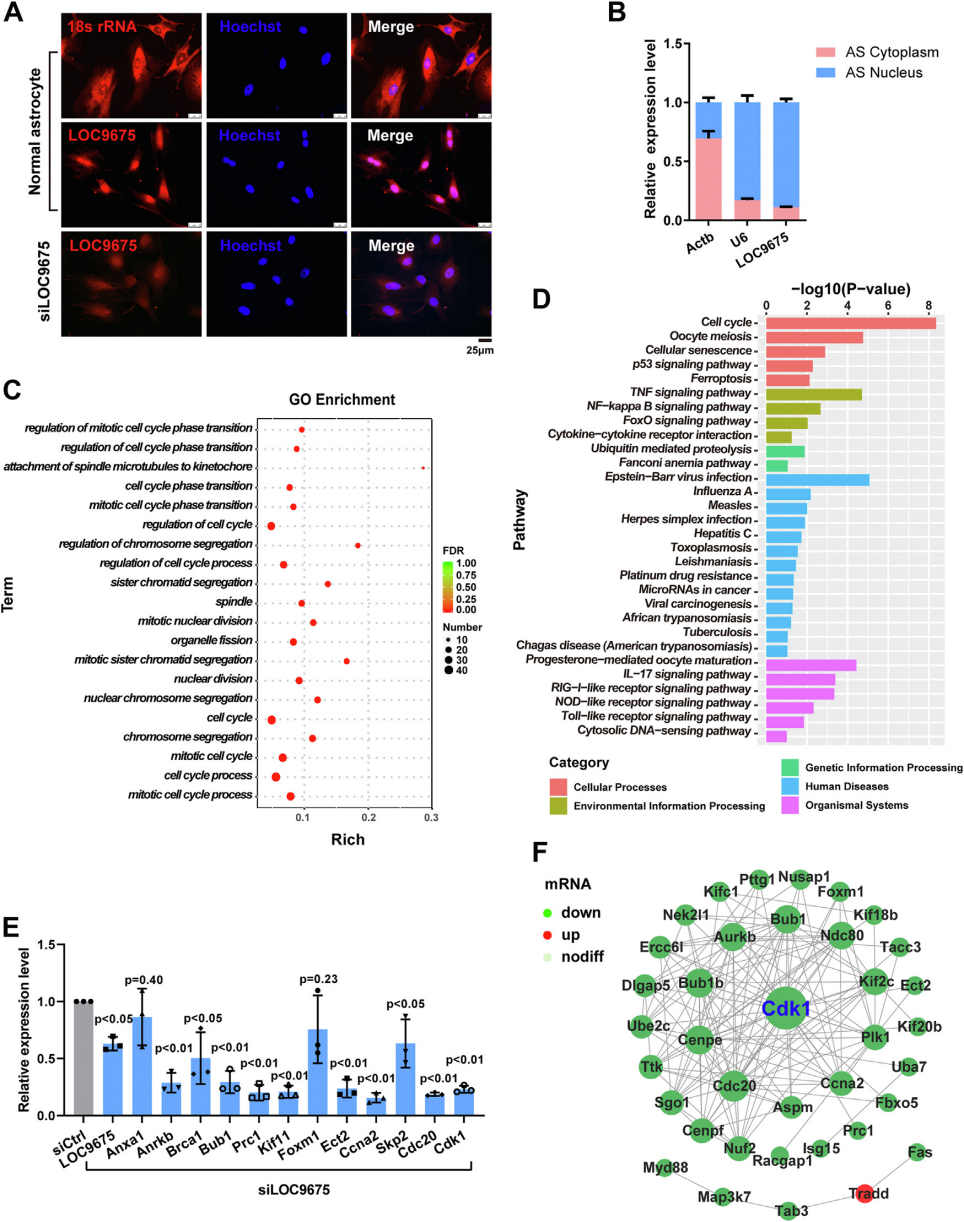
**Cellular localization of LOC9675 in astrocytes and gene expression profile alteration after LOC9675 knockdown.***A*, representative FISH images showing that LOC9675 (*red*) mostly localized to the astrocyte nucleus. The nuclei were stained with DAPI (*blue*); 18S rRNA molecules, in the cytoplasm, were used as a positive control, the scale bar represents 25 μm. *B*, relative expression levels of LOC9675 in the cytoplasm and nuclei of astrocytes. The relative levels of *Actb* expression in the cytoplasm and nucleus of astrocytes were 70% and 30%, respectively. The relative levels of *U6* expression in the cytoplasm and nucleus of astrocytes were 17% and 83%, respectively. The relative levels of LOC9675 expression in the cytoplasm and nucleus of astrocytes were 11% and 89%, respectively. The data are shown as the mean ± SD, n = 3. *C*, top 20 Gene Ontology (GO) terms from the GO enrichment analysis of target genes using LOC9675. GO analysis showed that many of the altered genes were associated with the cell cycle. *D*, Kyoto Encyclopedia of Genes and Genomes (KEGG) analysis. KEGG analysis showed the most potential signaling pathways involving the differentially expressed genes were cell cycle. *E*, twelve of these downregulated mRNAs were chosen for validation by qRT-PCR analysis, and the results were similar to those obtained from RNA-seq. The data are shown as the mean ± SD. Data were analyzed using one-way ANOVA followed by Tukey’s post hoc test (*p* < 0.05). Unpaired Student's *t* test was used to compare two groups (*versus* siCtrl, n = 3). The *p* values are shown on the panel. *F*, protein‒protein interaction (PPI) network of LOC9675 knockdown. Nodes correspond to the genes and edges of the PPI. DAPI, 4′,6-diamidino-2-phenylindole; qRT-PCR, quantitative reverse transcription.

Next, we investigated the possible mechanisms through which LOC9675 regulates astrocyte proliferation. lncRNAs play different roles depending on their localization in the cytoplasm or the nucleus. We first determined the cellular localization of LOC9675. FISH assays showed that LOC9675 mostly accumulated in the nuclei of astrocytes, while 18S rRNA was detected in the cytoplasm and nuclei. We then performed the FISH assay using siLOC9675 and found that the positive signals significantly decreased in nuclei (Fig. 3*A*). We also performed cytoplasmic/nuclear RNA separation and real-time RT-PCR, which revealed that LOC9675 transcripts were mainly located in the nucleus (Fig. 3*B*), similar to U6 expression.

The lncRNAs in the nucleus usually regulate gene transcription (19). Therefore, the gene expression profiles altered by siLOC9675 were analyzed using RNA-seq. A total of 1457 mRNAs including 705 upregulated and 752 downregulated mRNAs (the list of the genes is shown in Table S2) were significantly differentially expressed in siLOC9675 treated astrocytes (Fig. S2). Gene Ontology (GO) enrichment analysis showed that differentially expressed genes (DEGs) were mostly related to the mitotic cell cycle (Fig. 3*C*), whereas the most enriched Kyoto Encyclopedia of Genes and Genomes (KEGG) pathway was the cell cycle (Fig. 3*D*). The mRNA expression of several cell cycle-related genes was verified. As shown in Figure 3*E*, compared with the control, the mRNA levels of *Anrkb*, *Brca1*, *Bub1*, *Prc1*, *Kif11*, *Ect2*, *Ccna2*, *Skp2*, *Cdc20*, and cyclin-dependent kinase (Cdk)1 were significantly decreased in siLOC9675-treated astrocytes. These results are consistent with their expression in our RNA-seq data. Protein–protein interaction analysis of the DEGs (Fig. 3*F*) revealed that CDK1 was the most prominent core gene.

### Depletion of LOC9675 inhibited astrocyte proliferation by decreasing CDK1 expression

Our study results indicate that depletion of LOC9675 inhibits astrocyte proliferation by regulating cell cycle-related gene expression and that CDK1 is a hub gene candidate. We investigated how LOC9675 regulates CDK1 expression in astrocytes.

**Figure 4:**
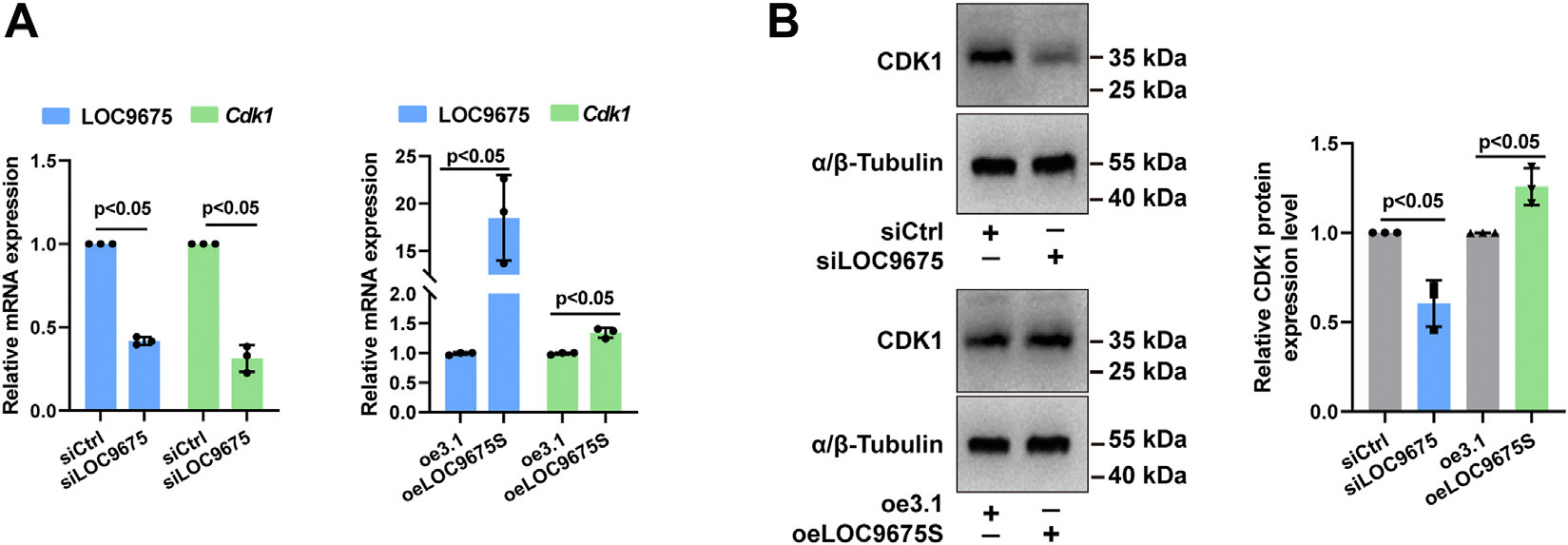
**Depletion of LOC9675 inhibited astrocytes proliferation *via* decreasing CDK1 expression.***A*, real-time quantitative PCR results for LOC9675 and Cdk1 after LOC9675 siRNA (*left panel*) or overexpression (*right panel*). The data are shown as the mean ± SD. Data were analyzed using unpaired Student’s *t* test (*versus* control, n = 3). The *p* values are shown on the panel. *B*, protein expression levels of CDK1 after LOC9675 siRNA or overexpression. *Left panel*: representative Western blot results. *Right panel*: statistical analysis. The data are shown as the mean ± SD. Data were analyzed using unpaired Student’s *t* test (*versus* control, n = 3). The *p* values are shown on the panel. CDK, cyclin-dependent kinase.

As shown in Figure 4*A*, compared to the control group, depletion of LOC9675 resulted in a 68.6% decrease and overexpression of LOC9675 resulted in a 33.8% increase in *Cdk1* mRNA levels. The protein levels were consistent with the mRNA levels (Fig. 4*B*). Since CDK1 overexpression is associated with the promotion of cell proliferation, we evaluated the relationship between LOC9675 and CDK1 during astrocyte proliferation. The results revealed that LOC9675 depletion significantly decreased CDK1 expression and notably inhibited astrocyte proliferation, as the percentage of EdU-positive cells was reduced (Fig. 1*E*). We investigated the mechanism by which LOC9675 regulates CDK1. Based on LOC9675 localization in nuclei, we propose that it regulates *Cdk1* transcription.

### LOC9675 directly interacts with CTCF, a novel transcription factor of the CDK1 gene

**Figure 5:**
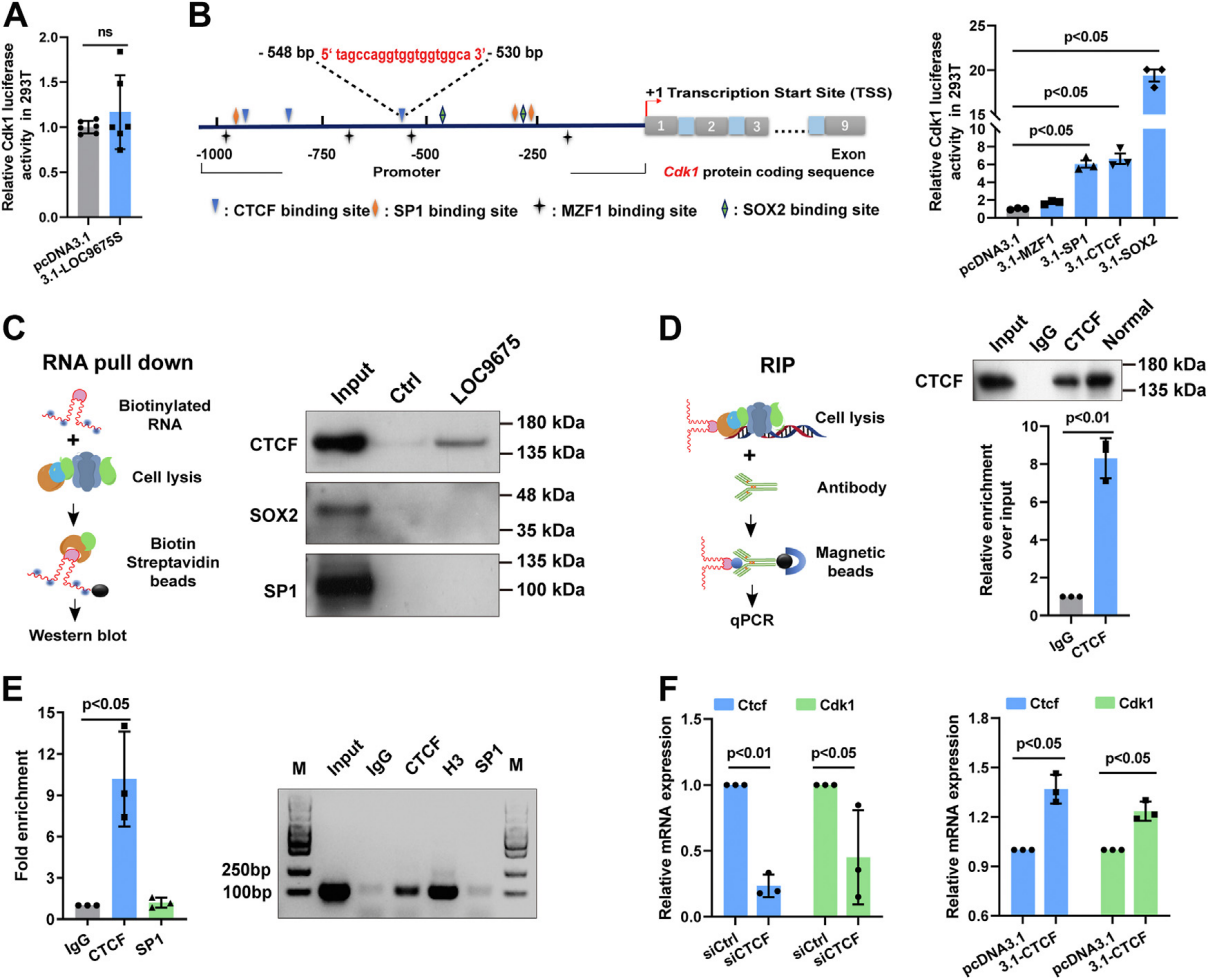
**LOC9675 directly interacted with CTCF, a novel transcription factor of the *Cdk1* gene.***A*, relative luciferase activity (firefly luciferase activity divided by Renilla luciferase activity) in the presence of pcDNA3.1 or pcDNA3.1-LOC9675S with *Cdk1* gene promoter plasmid overexpression in HEK293T cells after normalization to the relative luciferase activity of the pcDNA3.1(+) control. The data are shown as the mean ± SD. Data were analyzed using unpaired Student’s *t* test (*versus* pcDNA3.1, n = 6). The *p* value is shown on the panel. *B*, *left panel*, diagrammatic sketch of the possible binding sites of the CTCF, SP1, MZF1, and SOX2 transcription factors predicted within −1000 bp to +1 bp of the *Cdk1* gene promoter. *Right panel*, results of luciferase reporter analysis after pcDNA3.1, pcDNA3.1-CTCF, 3.1-SP1, 3.1-MZF1, and 3.1-SOX2 plasmid overexpression with the *Cdk1* gene promoter plasmid. The data are shown as the mean ± SD. Data were analyzed using one-way ANOVA followed by Tukey’s post hoc test (*p* < 0.05). Unpaired Student's *t* test was used to compare two groups (*versus* pcDNA3.1, n = 3). The *p* values are shown on the panel. *C*, *left panel*: schematic representation of the RNA pull-down assay. *Right panel*: Western blot (WB) confirmed the association of LOC9675 with CTCF but not with SP1 or SOX2 proteins. Twenty micrograms of cell lysate was used as the input. *D*, *left panel*: schematic of the RNA immunoprecipitation (RIP) assay. *Right panel*: qRT-PCR detection of retrieved RNAs. The data are shown as the mean ± SD. Data were analyzed using unpaired Student’s *t* test (*versus* IgG, n = 3). The *p* value is shown on the panel. *E*, representative results of ChIP analysis. The anti-CTCF/H3/SP1 antibody immunoprecipitated a CTCF/H3/SP1-DNA complex; the DNA region bound by CTCF/H3/SP1 was identified by real-time quantitative PCR (qPCR) (*left*) or conventional PCR (*right*) using *Cdk1*-specific primers, and IgG was used as an immunoprecipitated control. The data are shown as the mean ± SD. Data were analyzed using one-way ANOVA followed by Tukey’s post hoc test (*p* < 0.05). Unpaired Student's *t* test was used to compare two groups (*versus* IgG, n = 3). The *p* values are shown on the panel. *F*, qPCR results for *Ctcf* and *Cdk1* after Ctcf siRNA (*left panel*) or overexpression (*right panel*) of astrocytes for 24 h. The data are shown as the mean ± SD. Data were analyzed using unpaired Student’s *t* test (*versus* control, n = 3). The *p* values are shown on the panel. CDK, cyclin-dependent kinase; ChIP, chromatin immunoprecipitation; CTCF, CCCTC-binding factor; IgG, immunoglobulin G; pcDNA, plasmid cloning DNA; qRT-PCR, quantitative reverse transcription.

A dual-luciferase reporter assay was performed in HEK293T cells. The results (Fig. 5*A*) showed that LOC9675 overexpression did not alter *Cdk1* at the transcriptional level. This result suggests that LOC9675 regulates CDK1 *via* certain transcription factors (TFs). We then searched the JASPAR and AnimalTFDB (v3.0) databases and used RPISeq online software to predict TF binding to the rat *Cdk1* promoter region covering −1000 bp to +1 bp (Fig. S3). As shown in Figure 5*B*, 4 TFs, CCCTC-binding factor (CTCF), MZF1, SP1, and SOX2 were selected for further analysis. A dual-luciferase reporter assay was performed to determine whether these four TFs regulated CDK1 gene expression. The data showed that CTCF, SP1, and SOX2 significantly upregulated *Cdk1* transcription, whereas MZF1 had no effect (Fig. 5*B*, right panel). Next, we performed an RNA pull-down assay in astrocytes, and the results revealed that the CTCF protein could be pulled down by LOC9675 but not by SP1 or SOX2 (Fig. 5*C*). We further performed RNA immunoprecipitation (RIP) assays in astrocytes, and the results showed that LOC9675 was enriched in the complex pulled down by the CTCF protein (Fig. 5*D*). These results revealed that LOC9675 directly interacts with CTCF.

Finally, we performed a chromatin immunoprecipitation (ChIP) assay in astrocytes, and as shown in Figure 5*E*, the results suggested that the transcription factor CTCF could bind to the predicted region (Fig. 5*B*, left panel, red characters, −548 bp to −530 bp) in the *Cdk1* promoter. Next, we tested whether CTCF affected CDK1 transcription by overexpressing and depleting CTCF in astrocytes. As shown in Figure 5*F*, after CTCF siRNA treatment, *Cdk1* mRNA was reduced by 54.9% (Fig. 5*F*, left panel), while when CTCF was overexpressed, the mRNA level of *Cdk1* showed a 23.5% increase (Fig. 5*F*, right panel). Taken together, these results suggest that CTCF is a novel positive regulator of *Cdk1* gene transcription.

### The cooperation of CTCF and LOC9675 could regulate CDK1 transcription and thereby strictly control astrocyte proliferation

The above results revealed that lncRNA LOC9675 did not itself affect *Cdk1* gene transcription but directly interacted with the CTCF protein. We further investigated how the LOC9675-CTCF interaction affected the mRNA level of *Cdk1*. We hypothesized that the interaction between LOC9675 and CTCF regulates *Cdk1* transcription in astrocytes.

**Figure 6:**
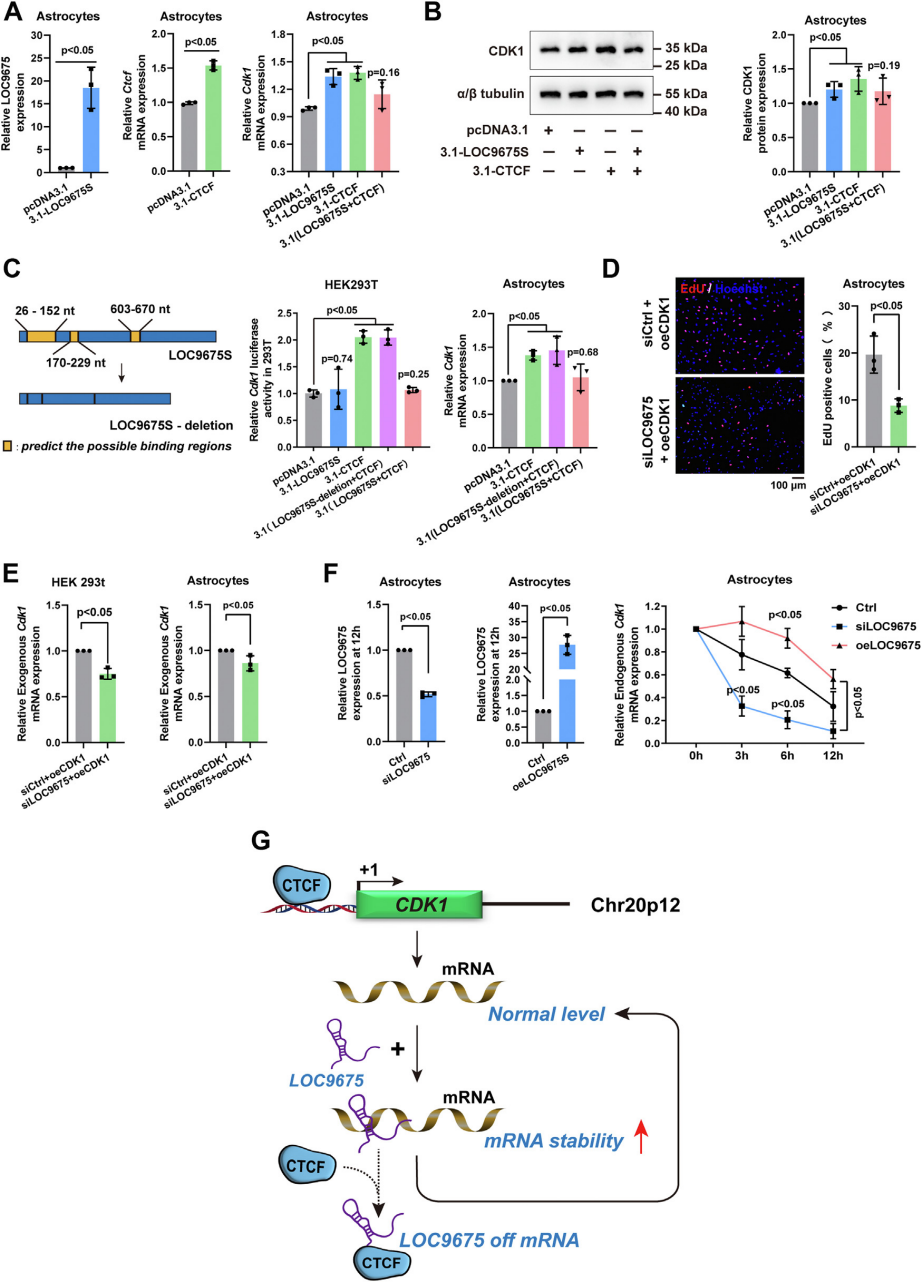
**LOC9675 interacting with CTCF contributed to astrocyte proliferation by regulating CDK1 expression.***A*, qPCR results of LOC9675, *Ctcf*, and *Cdk1* after pcDNA3.1-LOC9675S, 3.1-CTCF, and 3.1(LOC9675S+CTCF treatment for 24 h in astrocytes. The data are shown as the mean ± SD. Data were analyzed using one-way ANOVA followed by Tukey’s post hoc test (*p* < 0.05). Unpaired Student's *t* test was used to compare two groups (*versus* pcDNA3.1, n = 3). The *p* values are shown on the panel. *B*, Western blotting results of CDK1 in astrocytes after different treatments. The data are shown as the mean ± SD. Data were analyzed using one-way ANOVA followed by Tukey's post hoc test (*p* < 0.05). Unpaired Student's *t* test was used to compare two groups (*versus* pcDNA3.1, n = 3). The *p* values are shown on the panel. *C*, *left panel*, schematic diagram of LOC9675S mutant plasmid synthesis. *Middle panel*, the relative luciferase activity (firefly luciferase activity divided by Renilla luciferase activity) in the presence of pcDNA3.1, pcDNA3.1-LOC9675S, pcDNA3.1-CTCF, pcDNA3.1-LOC9675S(deletion)+CTCF, and pcDNA3.1-LOC9675S+CTCF with *Cdk1* gene promoter plasmid overexpression in HEK293T cells. Luciferase activity was normalized to that of control cells transfected with pcDNA3.1(+). *Right panel*, qPCR results for *Cdk1* after pcDNA3.1 3.1-CTCF, 3.1(LOC9675S+CTCF), and 3.1(LOC9675S-deletion+CTCF) treatment for 24 h in astrocytes. The data are shown as the mean ± SD. Data were analyzed using one-way ANOVA followed by Tukey's post hoc test (*p* < 0.05). Unpaired Student's *t* test was used to compare two groups (*versus* pcDNA3.1, n = 3). The *p* values are shown on the panel. *D*, 5-ethynyl-2′- (EdU) results after siCtrl+oeCDK1 and siLOC9675+oeCDK1 treatment for 48 h in astrocytes. *Left panel*: representative EdU images; the scale bar represents 100 μm. *Right panel*: statistical analysis. The data are shown as the mean ± SD. Data were analyzed using unpaired Student’s *t* test (*versus* siCtrl+oeCDK1, n = 3). The *p* value is shown on the panel. *E*, qPCR results for exogenous *Cdk1* after siCtrl+oeCDK1 and siLOC9675+oeCDK1 treatment for 24 h in HEK293T cells (*left panel*) and astrocytes (*right panel*). The data are shown as the mean ± SD. Data were analyzed using unpaired Student’s *t* test (*versus* siCtrl+oeCDK1, n = 3). The *p* value is shown on the panel. *F*, qPCR results of LOC9675 in the siLOC9675 group (*left panel*) and oeLOC9675S group (*middle panel*) after 2 μg/ml actinomycin D treatment for 12 h in astrocytes. The qPCR results of endogenous *Cdk1* in the Ctrl group, siLOC9675 group, and oeLOC9675S group after 2 μg/ml actinomycin D treatment for 3 h, 6 h, and 12 h in astrocytes (*right panel*). The data are shown as the mean ± SD. Data were analyzed using unpaired Student’s *t* test or two-way ANOVA followed by Bonferroni's post hoc test (*versus* control, n = 3). The *p* values are shown on the panel. *G*, diagram of cooperation between CTCF and LOC9675, which controls *Cdk1* transcription at a steady level. CDK, cyclin-dependent kinase; CTCF, CCCTC-binding factor; pcDNA, plasmid cloning DNA.

Therefore, *Cdk1* transcript was examined after CTCF and LOC9675 overexpression in spinal astrocytes. As shown in Figure 6*A*, either LOC9675 or CTCF overexpression increased *Cdk1* transcript, whereas the simultaneous overexpression of LOC9675 and CTCF restored *Cdk1* expression to the control level. The protein levels tended to be consistent with the mRNA levels (Fig. 6*B*).

This unexpected result suggests that LOC9675 and CTCF have no synergistic effect on *Cdk1* transcription. To verify this, we performed a dual-luciferase reporter assay in HEK293T cells to explore the effect of CTCF and LOC9675 on the transcriptional activity of *cis*-elements in the Cdk1 promoter. In addition, we designed and generated a mutant LOC9675 that lacks the 255 nt fragment that bound to CTCF. The results showed that normal LOC9675 restored *Cdk1* transcription increased by CTCF; however, mutant LOC9675 had no such rescue effect (Fig. 6*C*, middle panel). A similar phenomenon was observed in astrocytes (Fig. 6*C*, right panel). These data indicate that LOC9675 may regulate *Cdk1* mRNA stabilization posttranscriptionally.

To test this hypothesis, we examined the effect of LOC9675 on exogenous *Cdk1* mRNA. The pEGFP-N1-Cdk1 plasmid containing *Cdk1* cDNA was introduced into the astrocytes along with siLOC9675. The percentage of EdU-positive cells (Fig. 6*D*) revealed a notable decrease in astrocyte proliferation after the depletion of LOC9675. Next, we detected the mRNA levels of exogenous *Cdk1* using qRT-PCR with one primer in the pEGFP-N1 vector and another within the coding sequence region (Table 1). The results (Fig. 6*E*) showed that the mRNA expression of exogenous *Cdk1* significantly decreased in both HEK293T cells and astrocytes. These data suggest that LOC9675 affects the stability of *Cdk1* mRNA molecules.

**Table 1:** The sequences of primers and siRNAs

Name	Sense (5′-3′)	Antisense (5′-3′)
*Gapdh*	tgccactcagaagactgtgg	caacggatacattgggggta
*LOC9675*	tgaacaaaggacggactggt	cttcggttctcctggtgact
*Anxa1*	aaaggggacagacgtgaatg	tggcacacttcacaatggtt
*Aurkb*	cataaagcccgagaacctgc	gcatccgcccttcaatcatc
*Brca1*	tacaagagcagcccttcaca	ttgcttggtcatttggctcc
*Prc1*	tcccaacaagtgtggcaaag	tgactactgacttgctgcca
*Bub1*	tccagccatcaagaccaaga	ctttccatcagctgcatccc
*Kif11*	tggacgttcacaaagcactg	gctgctaacgactgctcttc
*Foxm1*	ttagctcatacctggtgccc	cttcgttggatagcagcacc
*Ect2*	tccagttcctccaaagcagt	gtcttgatttcctcgggtgc
*Ccna2*	tacccagtacttcctgcacc	tgtaccaatgactcaggcca
*Skp2*	cgatgagtctctctggcagt	gctgtacacggaaagagctg
*Cdk1*	ggccagaagtagagtccctg	tatgggtgcttaagggccat
Exogenous-*Cdk1*	tagtgaaccgtcagatccgc	ttttcttcatggccacgatc
*Cdc20*	ctcaggctcagtggaaaacc	gatttcaggggcatcaagaa
*ACTB*	ttgtgatggactccggagac	aatgtcacgcacgatttccc
*Mki67*	cgactcaagagactgggtgt	ctgggtcttgtttctgtgcc
*Ctcf*	ccagtgcagtttgtgcagtt	gtggggacaatgaaatttgg
*Ctcf* Chip	gttctatcactgggctaaatctcc	agtgcccaggttaaaggtgc
*SNHG26*	gggccagttgtctcagaatc	cttgtccagctgcaaaatca
*Lnc-5′ RACE*	ctaatacgactcactatagggcaagcagtggtatcaacgcagagt	
*Lnc-3′ RACE*	cgaaagcgacaaggccgtgatcccgaaagcttttttttttttttttttttttttttvn	
Control (Ctrl) siRNA	uucuccgaacgugucacgutt	acgugacacguucggagaatt
LOC9675 siRNA-1	gcgcggacaucgccuauuutt	aaauaggcgauguccgcgctt
LOC9675 siRNA-2	ccaggaugauagugaauuutt	aaauucacuaucauccuggtt
LOC9675 siRNA-3	ggagaaccgaagauguacutt	aguacaucuucgguucucctt
CTCF siRNA	gcccauuaauauaggagaatt	uucuccuauauuaaugggctt
SNHG26 siRNA-1	gggagguuaugccaccautt	augguggcauaauccuccctt
SNHG26 siRNA-2	gccgaauguuguagcugautt	aucagcuacaacauucggctt

Finally, we used actinomycin D, a transcription inhibitor, to confirm this hypothesis in astrocytes. After transfection with control, siLOC9675 RNA, and oeLOC9675 plasmid for 24 h, actinomycin D was added. Total RNA was isolated at 0, 3, 6, and 12 h after actinomycin D addition. The qRT-PCR results showed that in the control sample, treatment with actinomycin D resulted in a decrease of 22.4%, 30.4%, and 67.7% in *Cdk1* mRNA levels (Fig. 6*F*), while siLOC9675 treatment led to a decrease of 67.4%, 79.4%, and 89.4% in *Cdk1* mRNA levels, and oeLOC9675 led to decrease of - 6.7%, 8%, and 44.7% in *Cdk1* mRNA levels. These results verified that LOC9675 was directly involved in *Cdk1* mRNA stabilization.

Based on these data, we concluded that LOC9675 (shown in Fig. 6*G*) stabilizes *Cdk1* mRNA and increases CDK1 protein, thus contributing to cell proliferation, whereas the interaction of CTCF-LOC9675 undocks LOC9675 from *Cdk1* mRNA, thus maintaining *Cdk1* mRNA at a normal level.

## Discussion

The lncRNAs are extensively expressed and play multiple roles in gene regulation by interacting with DNA, RNA, and proteins. Mammalian genomes transcribe and generate thousands of lncRNAs, and in physiological and pathological states, the variety and quantity of lncRNAs are altered and involved in different biological processes, including SCI. Based on previous studies (16, 17), we noticed that many lncRNAs were altered after SCI, especially in the early stages when they showed drastic changes. We propose that epigenetic modulations respond quickly to alterations due to SCI. In this study, we examined one of the lncRNA molecules, LOC100909675 (LOC9675), which promptly increases after SCI, suggesting its potential role in regulating the early response to injury stimuli. As expected, specifically depriving LOC9675 of astrocytes reduced glial proliferation and facilitated axonal growth and motor recovery to a certain degree after SCI in rats.

Many studies have reported that during development and maturation, astrocytes gradually exit the cell cycle, and secrete neurotrophic factors to promote axonal growth (20, 21). We speculate that knockdown of LOC9675 decreases astrocyte proliferation and leads them to exit the cell cycle and mature. We analyzed the RNA-Seq data and found that the markers of immature astrocytes significantly decreased and those of mature astrocytes increased after the knockdown of LOC9675. Meanwhile, we performed ELISAs to detect the secreted brain-derived neurotrophic factor (BDNF) and NT-3 in the cultured astrocyte supernatant after siLOC9675 treatment and found that both BDNF and NT-3 increased, and BDNF showed a significant increase (Fig. S4). Therefore, based on our data, we conclude that the rapid increase in LOC9675 during the early stages of injury contributes to astrocyte proliferation.

In the nucleus, lncRNAs arrange chromatin structures and modulate gene transcription (8). As a nuclear-localized lncRNA, LOC9675 interacts with CTCF to regulate *Cdk1* transcription. Among the nine known CDKs (CDK1-CDK9) in vertebrates, CDK1 complexed with cyclins A and B is involved in cell mitosis (22). The modulation of CDK1-related antiapoptotic signals contributes to robust neuroprotection against SCI (23, 24). Cell proliferation is strictly regulated by Cdks with Cdk1 being the primary kinase involved in this process. It is a catalytic subunit of the highly conserved protein kinase complex, known as the mitosis-promoting factor, which is essential for the G1 to S phase and G to M phase transitions of the cell cycle (25, 26). CDK1 is regulated in multiple steps, including posttranslational modifications, miRNAs, and degradation (27); however, transcription remains the primary and crucial regulator. Our study provides an additional factor for *Cdk1* transcription regulation in addition to transcription factors.

In this study, we revealed that CTCF is a *Cdk1* gene transcription regulator. CTCF, a multifunctional transcription factor widely found in eukaryotes, is a highly conserved zinc-finger protein. On the one hand, CTCF acts as a classical positive or negative transcription factor when it binds to the promoter of target genes. On the other hand, CTCF can be an insulator protein by binding to the chromatin insulator domain, which could prevent the interaction of the promoter of the target gene with its enhancer or silencer (28). Recent studies have reviewed CTCF as a regulator of noncoding transcription, suggesting that CTCF deregulation results in an epigenetic imbalance during both development and disease (29). To date, publications have suggested that CTCF performs various functions that are somewhat contradictory; however, researchers consider CTCF to play a crucial role in creating boundaries between topologically associated domains in chromosomes. Within these domains, CTCF facilitates interactions between transcriptional regulatory sequences (30, 31).

Our study indicated that CTCF is a *trans*-acting factor that facilitates *Cdk1* transcription. The lncRNA LOC9675 interacts with CTCF to regulate CDK1 expression, thereby regulating astrocyte proliferation, either *in vitro* or *in vivo*. Interestingly, when CTCF and LOC9675 were simultaneously upregulated, the *Cdk1* transcript level stabilized at the normal level. As an important regulator of the cell cycle, CDK1 is controlled scrupulously (32, 33). lncRNAs are involved in the regulation of mRNA stability (34, 35, 36). Our data revealed an interacting regulatory model of LOC9675-CTCF-CDK1. This model (Fig. 6*G*) could reasonably explain that under normal conditions, astrocyte proliferation is strictly modulated. Excess LOC9675 stabilized *Cdk1* mRNA and increased cell proliferation, and CTCF overexpression removed LOC9675 from *Cdk1* mRNA by interaction, thus restoring it to the normal transcriptional level.

We tried to extend the understanding of LOC9675 function in glioma proliferation, following the online database (UCSC Genome Browser Gateway, http://genome-asia.ucsc.edu/cgi-bin/hgGateway?redirect=manual&source=genome.ucsc.edu), and in accordance with a previous publication (37), we found that the lncRNA SNHG26 is the homologous gene of LOC9675 with the highest similarity in the human genome. SNHG26 is related to tumor proliferation (38). We tested SNHG26’s role in the human glioma cell line, U251. The results are shown in Fig. S5, the knockdown of SNHG26 significantly reduced the proliferation and migration of U251 cells and significantly reduced the expression of CDK1 at both the mRNA and protein levels. These results indicated that lncRNA LOC9675 or SNHG26 conservatively regulated *Cdk1* transcription.

## Experimental procedures

### Animals and SCI procedure

T9 lateral hemisection was performed as described previously (17). Adult SD rats weighing 240 to 260 g were obtained from the Animal Center of Nantong University. Rats were randomly divided into eight groups corresponding to 0 h, 3 h, 6 h, 12 h, 1 day, 7 days, 14 days, 28 days, 42 days, and 56 days postinjury with three rats per group. Similarly, the sham operation groups were established at the same time points. All the studies reported here were submitted to the Ethics Committee on Animal Experimentation of Nantong University, and all procedures were approved (S20200330-003) by the Animal Care and Use Committee of Nantong University. Efforts were made to minimize the number of animals used and their suffering.

### RNA extraction and real-time quantitative PCR

Total RNA was extracted using TRIzol reagent (Thermo Fisher Scientific, # 15596-026), and cDNA was synthesized using the HiScript III 1st Strand cDNA Synthesis Kit (+ gDNA wiper) (Vazyme, # R312-02). PCR was performed using gene-specific primers for *Gapdh* expression to control mRNA integrity. qPCR was performed using AceQ qPCR SYBR Green Master Mix (with high ROX) (# Q141-02; Vazyme) on a Step One Plus Real-time PCR System (Applied Biosystems). Primers were synthesized by Thermo Fisher Scientific. *Gapdh* was used as the internal control. The primers used in this study are listed in Table 1.

### Primary astrocytes culture and siRNA transfection

Primary astrocytes of postnatal day one rat spinal cord were prepared as we previously described (39); they were cultured in Dulbecco's Modified Eagle Medium/Nutrient Mixture F-12 (DMEM/F12, Invitrogen, # 11320033), supplemented with 10% fetal bovine serum (Gibco, # 10099141C), 0.5 mM glutamine (Gibco, # 25030164), and 1% penicillin-streptomycin (Gibco, # 15140122). Subsequently, they were incubated in a humidified atmosphere of 95% air and 5% CO_2_ at 37 °C. When cells became confluent, the cultures were shaken at 150 rpm for 16 h for purification. Purified astrocytes at passage 2 were used for siRNA transfection at a final concentration of 200 nM using a NEPA21 electrical transfection instrument (40). siRNAs were synthesized by GenePharma. The sequences are listed in Table 1.

### Primary neurons culture and axonal length analysis

E14 spinal neurons were isolated from day 14 embryos of pregnant SD rats as described previously (41). Cultured neurons were fixed in a buffer containing 4% paraformaldehyde, 0.2% glutaraldehyde, 1 × PHEM (60 mM Pipes, 25 mM Hepes, 10 mM EGTA, and 2 mM MgSO_4_), and 0.1% Triton X-100 for 25 min. Cultures were then washed with PBS thrice and blocked with 10% goat serum containing 10 mg/ml bovine serum albumin for 1 h. Cultures were incubated with mouse anti-Tuj1 antibody (Biolegend, # 801201) overnight at 4 °C. On the next day, cultures were rewarming for 30 min, rinsed with PBS, and incubated with cy3-conjugated goat anti-mouse IgG (Jackson ImmunoResearch) at room temperature (22 °C) for 2 h. The cells were counterstained with Hoechst stain (Sigma-Aldrich, # B2261). The cells were then washed with PBS and mounted in antifade mounting medium. Fluorescent images were acquired using an Axio Imager M2 fluorescence microscope (Zeiss). For axonal length analysis, images were acquired at 20× magnification using a Zeiss microscope (Axio Imager M2; Carl Zeiss AG) and analyzed using the IPP software: “(http://www.totalsmart.com.tw/en/image-pro-plus). Astrocyte activation and proliferation contribute to glial scar formation during spinal cord injury (SCI), which limits nerve regeneration. The long noncoding RNAs (lncRNAs) are involved in astrocyte proliferation and act as novel epigenetic regulators. Here, we found that lncRNA-LOC100909675 (LOC9675) expression promptly increased after SCI and that reducing its expression decreased the proliferation and migration of the cultured spinal astrocytes. Depletion of LOC9675 reduced astrocyte proliferation and facilitated axonal regrowth after SCI. We used RNA-seq to analyze gene expression profile alterations in LOC9675-depleted astrocytes and identified the cyclin-dependent kinase 1 (*Cdk1*) gene as a hub candidate. Our RNA pull-down and RNA immunoprecipitation assays showed that LOC9675 directly interacted with the transcriptional regulator CCCTC-binding factor (CTCF). Dual-luciferase reporter and chromatin immunoprecipitation assays, together with down/upregulated expression investigation, revealed that CTCF is a novel regulator of the Cdk1 gene. Interestingly, we found that with the simultaneous overexpression of CTCF and LOC9675 in astrocytes, the *Cdk1* transcript was restored to the normal level. We then designed the deletion construct of LOC9675 by removing its interacting region with CTCF and found this effect disappeared. A transcription inhibition assay using actinomycin D revealed that LOC9675 could stabilize *Cdk1* mRNA, while LOC9675 depletion or binding with CTCF reduced *Cdk1* mRNA stability. These data suggest that the cooperation between CTCF and LOC9675 regulates *Cdk1* transcription at a steady level, thereby strictly controlling astrocyte proliferation. This study provides a novel perspective on the regulation of the *Cdk1* gene transcript by lncRNA LOC9675.” We defined an axon as a cell with a neurite length >20 μm, according to our recent publication (42).

### Cell proliferation and migration assays

We used the EdU (Ribobio) incorporation assay to test cell proliferation according to our previous study (17). At 24 h posttransfection, the astrocytes were digested and counted; 5 × 10^4^ astrocytes were plated onto 0.01% poly-L-lysine-coated 24-well plates. At the indicated time points, 50 mM EdU was added to the cells, which were incubated for 2 h. After being fixed, the cells were analyzed using a Cell-Light EdU Apollo567 *In Vitro* Imaging Kit (RiboBio, # C10310-1). Cell proliferation was expressed as the ratio of EdU-positive cells to total cells as determined from images of randomly selected fields obtained using a DMi8 fluorescence microscope (Leica Microsystems). In addition, CCK-8 (Vazyme Biotech, # A311-01) was used to test the cell viability according to the manufacturer’s protocol. Twenty-four hours after transfection, astrocytes were digested, counted, and plated in 96-well plates. CCK-8 (10 μl) was added at the indicated time points, and the plates were incubated for an additional 2 h. The absorbance at 450 nm was measured to determine cell viability.

For the Transwell migration assay, astrocytes were examined using 6.5 mm Transwell chambers with 8 μm pores (Costar). The 700 μl complete medium was added into the lower chambers, and a 200 μl sample of DMEM/F12 containing resuspended 5 × 10^4^ astrocytes was transferred to the top chamber, where the cells were allowed to migrate at 37 °C in 5% CO_2_. At specific time points, the upper surface of each membrane was cleaned using a cotton swab. Cells adhering to the bottom surface of each membrane were stained with 0.1% crystal violet, imaged, and counted using a DMi8 inverted microscope (Leica Microsystems). For the wound healing assay, 5 × 10^4^ astrocytes were seeded into the culture insert (Ibidi) in DMEM/F12 supplemented with 0.5% fetal bovine serum and 0.15 μg/ml mitomycin C (Sigma-Aldrich, # M5353) and incubated for 12 h. Afterward, the insert was extracted with tweezers, yielding a standardized wound of 500 μm. The dish was washed and imaged in the medium described above for 24 h. Wound closure was monitored and photographed at multiple sites and representative images were captured.

### RNA sequencing and bioinformatic analysis

Total RNA from astrocytes treated with control siRNA, and LOC9675 siRNA was collected and purified using a TruSeq Stranded mRNA LT sample preparation kit (Illumina Incaccording to the manufacturer’s instructions. Sequencing was performed at Shanghai Personal Biotechnology Co, Ltd. Transcript expression levels were estimated as fragments per kilobase per million reads values and quantified using HTSeq (0.9.1) p2. DEGs were designated with a threshold of *p* < 0.05 and a fold-change >2. For functional enrichment analysis, all DEGs were mapped to the KEGG database, and significantly enriched KEGG pathways (*p* < 0.05) were searched using a cluster profiler (3.16.1). GO enrichment was performed using topGO 2.40.0, and significantly enriched GO terms were defined using a hypergeometric test. The calculated *p*-value underwent false discovery rate correction, with a threshold of false discovery rate ≤ 0.05 applied.

### Fluorescence *in situ* hybridization and cell nucleus/cytoplasm fraction isolation

Cy3-labeled 18S rRNA and LOC9675 probes were designed and synthesized by RiboBio. RNA FISH was conducted using a fluorescent *in situ* Hybridization Kit (RiboBio Biotech, # C10910) according to the manufacturer’s instructions. The images were acquired using a DMi8 fluorescence microscope (Leica Microsystems).

Nuclear/cytoplasmic fractionation was performed using a Paris kit (Ambion, # AM1921) according to the manufacturer’s protocol. Briefly, P2 astrocytes were washed thrice with ice-cold PBS, and ice-cold CERI, CERII, and NER reagents were added sequentially. After vortexing and brief centrifugation, the supernatant was collected as the cytoplasmic fraction, and the remainder, with additional washing, was considered as the nuclear pellet.

### Rapid amplification of cDNA ends and plasmids constructs

Full-length amplification of lncRNA LOC9675 was performed with SMARTer RACE 5′/3′ Kit (Clontech, # 634858) according to the manufacturer’s instructions. The RACE primers are listed in Table 1., and the 1270 bp full-length sequence was determined to be LOC9675S (Fig. S6, 117 nt shorter than NR_110709 in 5′ end). For the LOC9675S deletion plasmid, we used the online website catRAPID (http://s.tartaglialab.com/page/catrapid_group) to predict possible binding regions, which were then eliminated (deletion 255 nt: 26–152 nt,170–229 nt, 603–670 nt; Fig. S7). LOC9675S plasmid, LOC9675S-deletion plasmid, Ctcf (NM_031824.1), Sox2 (NM_001109181.2), Sp1 (NM_012655.2), and Mzf1 (NM_001108470.1) plasmids were constructed using the pcDNA3.1 vector. Cdk1 (NM_019296.2) was constructed using the pEGFP-N1 vector. The plasmids were synthesized by General Biology.

### Dual-luciferase reporter assay and chromosome immunoprecipitation

For the reporter assay, Lipofectamine 3000 was used to cotransfect HEK293T cells with the plasmids. Luciferase activity was detected using a Dual-Luciferase Reporter Assay Kit (Promega, # E1910) 48 h posttransfection. Renilla luciferase activity was used as an internal control for firefly luciferase activity. As described in our previous study (43), a ChIP assay was performed using a SimpleChIP Enzymatic Chromatin IP Kit (Magnetic Beads) (CST, # 9005) according to the manufacturer’s instructions.

### RNA pull-down and RNA immunoprecipitation assay

Biotinylated RNAs were prepared using Pierce RNA 3′ End Desthiobiotinylation Kit (Thermo Fisher Scientific, # 20163) and T7/T3 RNA *in vitro* transcription kit (Ambion, # AM1308). The RNA pull-down assay was performed using the Pierce Magnetic RNA-Protein Pull-down Kit (Thermo Fisher Scientific, #20164) according to the manufacturer’s instructions. Approximately 50 pmol of total biotinylated RNAs were used for pull-down using 50 μl of streptavidin–magnetic beads. The cell lysate (150 μg) was incubated with the beads for 1 h at 4 °C with rotation. Bound proteins were retrieved by boiling at 100 °C with loading buffer and further analyzed by running 10% SDS-PAGE gel according to the Western blot standard protocol. RIP experiments were performed using an RNA Immunoprecipitation Kit (Gene-Seed, # P0101) following the manufacturer's instructions. RNAs was reverse-transcribed using a standard protocol and analyzed using qRT-PCR. The RIP assay was performed using rabbit anti-CTCF (CST, #3418S) and normal rabbit IgG (CST, #2729) antibodies.

### Adeno-associated virus injection

AAV9-gfaABC1D promoter-EGFP-Mir30 (shLOC9675)-WPRE-bGH PolyA (1.74 × 10^13^ vg/ml) and its vector virus AAV9-gfaABC1D promoter-EGFP-MCS-WPRE-bGH PolyA (1.75 × 10^13^ vg/ml) were purchased from GeneChem Biotech. We injected 3.0 μl of the virus into the T9 spinal cord at 0.2 μl/min using a glass micropipette (tip diameter, ∼20 μm) attached to a Nanoliter 2000 pressure injection apparatus (World Precision Instruments). The detailed injection procedure was as follows. There were five injection sites (Fig. 2*A*), and 0.6 μl was injected per point; 0.2 μl was injected at 1.5 mm under the spinal dura mater, and the needle was then slowly lifted by 1.2 mm to inject 0.2 μl and finally lifted by 0.9 mm to inject the residual 0.2 μl (39). The pipette was held in place for 2 min after each injection before being completely retracted from the spinal cord.

The procedure for tracing corticospinal neurons has been described previously (44). After anesthesia, the virus (pAAV2/9-SYN-mCherry-3FLAG [1.5 × 10^13^ vg/ml], OBIO) was injected into the T7 spinal cord bilaterally with a specially made thin glass needle at a rate of 0.25 μl/min and 0.75 μl/point to anterogradely trace the intrinsic spinal neuron using a rodent stereotaxic apparatus (RWD Life Science). To prevent backflow and allow viral diffusion, the needle was maintained in place for an additional 2 min after injection.

### BBB test

Behavioral recovery was scored in an open field using the BBB scale (45), where a score of 0 indicated complete paralysis and a score of 21 indicated complete mobility. Blind scoring ensured that the observers were unaware of the treatment received by the individual rats.

### Immunohistochemistry

For Immunohistochemistry, rats were perfused with 4% paraformaldehyde, and perilesional spinal cord tissues of 1.5 cm were collected and cryoprotected in 30% sucrose. Tissue sections (18 μm) were prepared by cryostat sectioning and treated with blocking buffer (0.4% bovine serum albumin, 5% goat serum, and 0.2% Triton-X 100 in PBS) for 60 min at room temperature. Primary antibodies for GFAP (Abcam, # ab4674, 1:200), mCherry (Abcam, # ab205402, 1:800), CSPG (Sigma-Aldrich, # C8035, 1:400), and Ki67 (Abcam, # ab16667, 1:400) were incubated overnight at 4 °C in blocking buffer. After rinsing with PBS, corresponding secondary antibodies were added and incubated for 120 min at room temperature. All tissues were counterstained with Hoechst (Sigma-Aldrich, # B2261, 1:4000). The tissues were washed and mounted. Fluorescence images were obtained using a fluorescence microscope (Zeiss).

### Western blotting analysis

Cells were lysed to extract total protein, and the protein concentrations in the total cell extracts were measured using a bicinchoninic acid protein assay. Total protein samples (10 μg) were separated by SDS-PAGE, and membranes were incubated overnight at 4 °C with the following primary antibodies: anti-CDK1 (Proteintech, #19532-1-AP), anti-α/β tubulin (Cell Signaling Technology, #2148S), anti-SP1(GeneTex, #GTX110593), anti-SOX2(Abcam, # ab97959) and anti-CTCF (CST, #3418S). After washing with Tris-buffered saline and 0.1% Tween 20, membranes were incubated with horseradish peroxidase-conjugated secondary anti-rabbit or anti-mouse antibodies at room temperature for 2 h. For visualization, the immunoreactive bands were treated with a chemiluminescent solution (Tanon, #180-5001) and detected using X-ray films. The optical density values of the target protein bands were quantified using Image J software (http://imagej.nih.gov/ij) and normalized to the α/β tubulin loading control.

### Actinomycin D treatment

Purified astrocytes at passage 2 were used for LOC9675 siRNA/plasmid transfection using a NEPA21 electrical transfection instrument and subsequently cultured for 24 h. To block transcription (35), 2 μg/ml actinomycin D (Medchemexpress, #HY-17559) was added to the cell culture medium after 24 h of transfection. After actinomycin D coculture at various time points, the remaining mRNA was detected using qRT-PCR.

### Statistical analysis

All data are expressed as mean ± standard (SD). Each experiment was repeated at least thrice. GraphPad Prism 8.3 software (GraphPad Software, https://www.graphpad.com/) was used for the data analysis. Statistical significance between datasets was tested using an unpaired two-tailed Student’s *t* test. One-way ANOVA was used to compare data from multiple groups. We applied two-way ANOVA to analyze the data affected by the two factors. A statistical significance was set at *p* < 0.05.

## Data availability

The data used to support the findings of this study are available from the corresponding author upon request.

## Supporting information

This article contains supporting information.

## Conflict of interest

The authors declare that they have no conflicts of interest with the contents of this article.
