# Supplementary material for: Cooperation of long noncoding RNA LOC100909675 and transcriptional regulator CTCF modulates Cdk1 transcript to control astrocyte proliferation
Source: J Biol Chem. 2023 Aug 9;299(9):105153. doi: 10.1016/j.jbc.2023.105153 (PMC10485634; doi:10.1016/j.jbc.2023.105153)

**Supporting Information:**


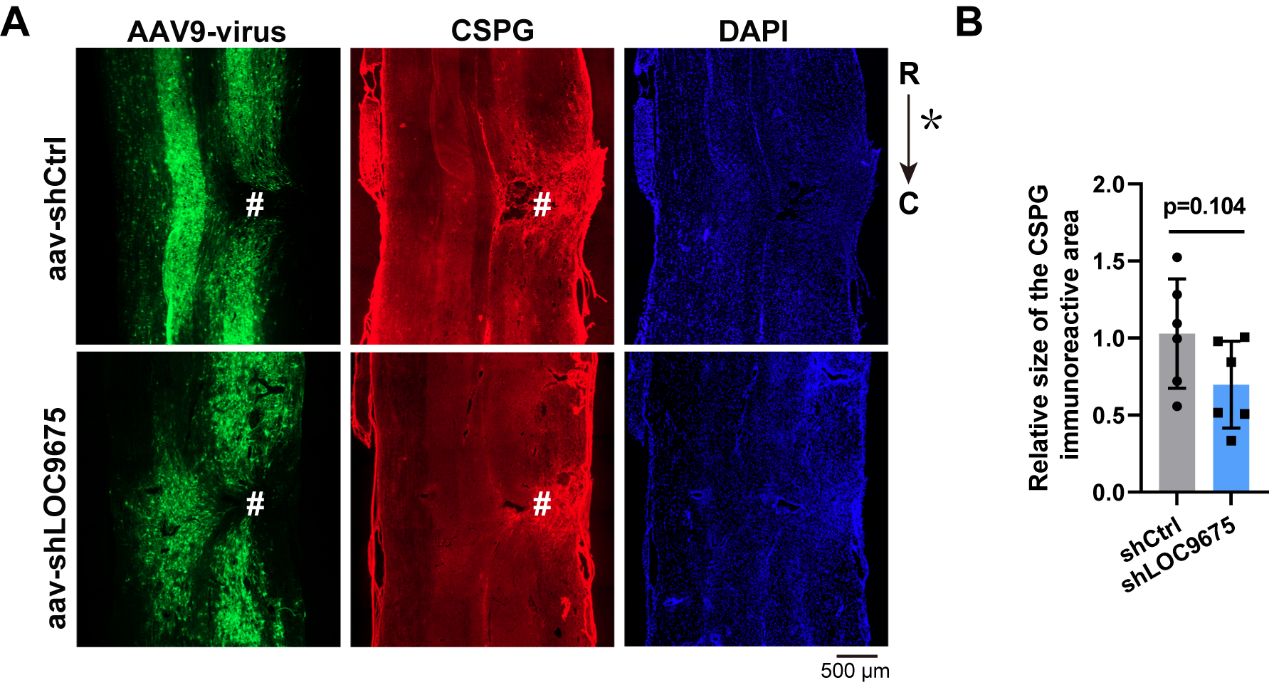


**Supplementary Figure S1. The expression of CSPG after AAV9-shCtrl or AAV9-shLOC9675 treatment in rats**

A, The representative immunofluorescence (IF) results showing the localization of the virus, CSPG, and DAPI at SCI 14 d. Scale bar = 500 μm. B, Statistical analysis. The injured site (labeled #); the asterisk represents the injured side, *R* indicates the rostral side, and *C* indicates the caudal side. The data are shown as the mean ± SD. Data were analyzed using unpaired Student’s t test (vs. aav-shCtrl, n = 6 rats). The *p* value is shown on the panel.


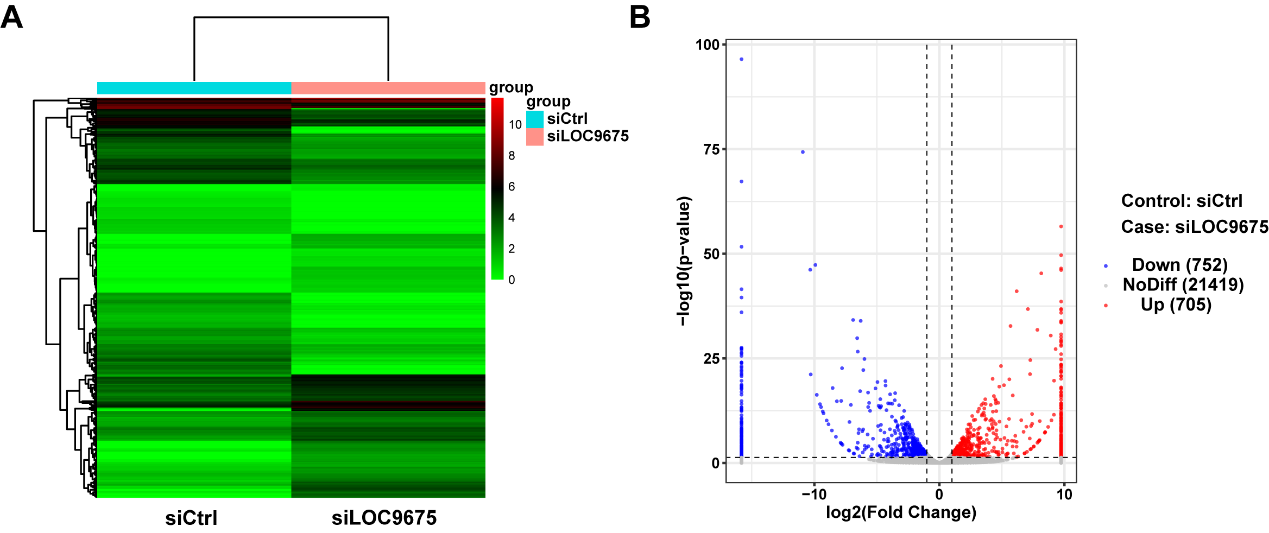


**Supplementary Figure S2. The heatmap (A) and a volcano plot (B) after siLOC9675 treatment in astrocytes**

**A**


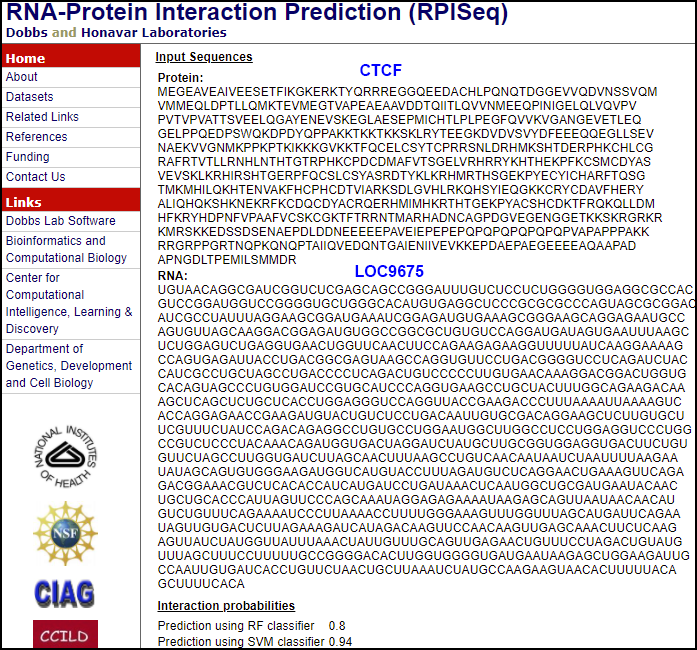


**B**


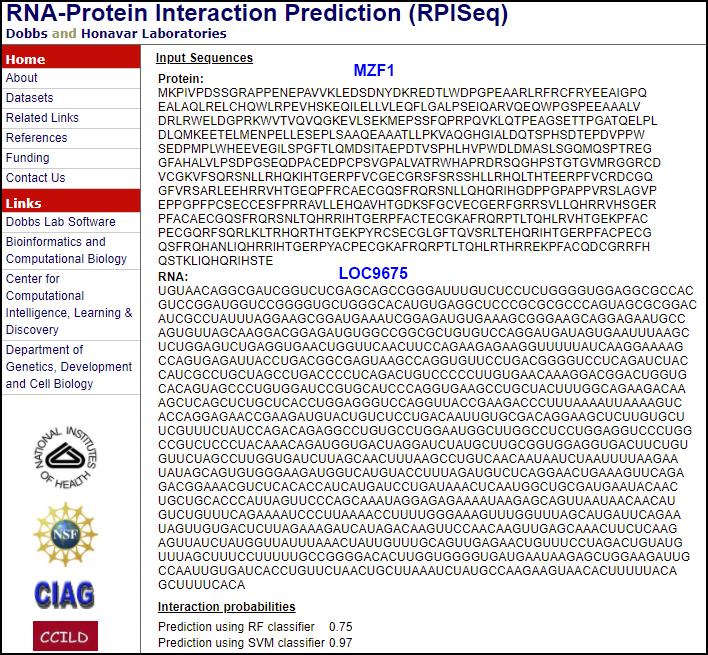


**C**


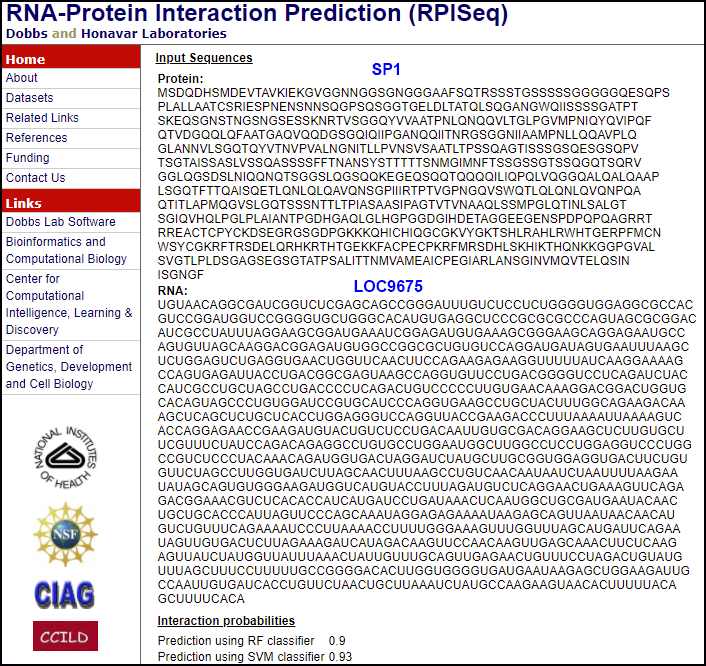


**D**


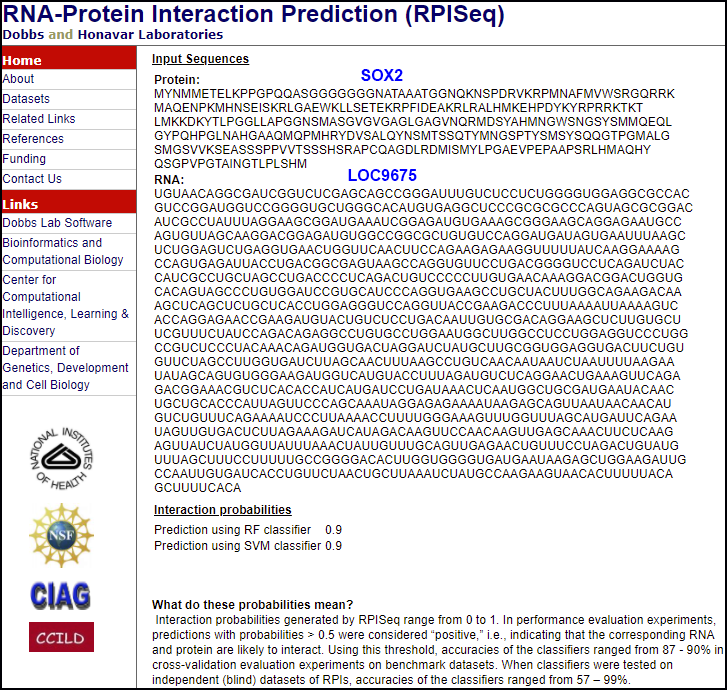


**Supplementary Figure S3. The results of lncRNA LOC9675 and CTCF(A)- MZF1(B)- SP1(C)-SOX2(D) protein interaction prediction**

***Enzyme-linked immunosorbent assay***

Control or LOC9675-knockdown astrocytes were seeded on 60-mm dishes at a density of 8 × 10^5^ cells/dish. After incubation for 48 hours, supernatants from the cell cultures were harvested and analyzed using the rat BDNF enzyme-linked immunosorbent assay (ELISA) kit (Cat: #HB500-Ra, Hengyuan Biotechnology Co., Ltd., Shanghai, China) and rat NT-3 ELISA kit (Cat: #HB372-Ra, Hengyuan Biotechnology Co., Ltd., Shanghai, China) following the manufacturer's instructions.


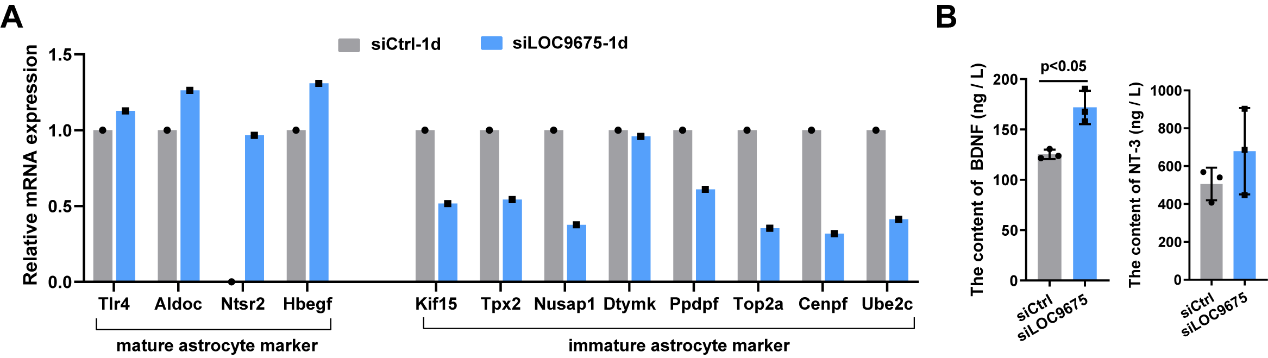


**Supplementary Figure S4. The expression pattern of markers of mature or immature astrocytes and the content of BDNF or NT-3 after siLOC9675 treatment**

A, The RNA-Seq data showed the alterations of immature or mature marker genes in siLOC9675 treated astrocytes.

B, The contents of BDNF and NT-3 secreted into the cultured astrocyte supernatant after siLOC9675 treatment. The data are shown as the mean ± SD. Data were analyzed using unpaired Student’s t test (vs. siCtrl, n = 3). The *p* values are shown on the panel.


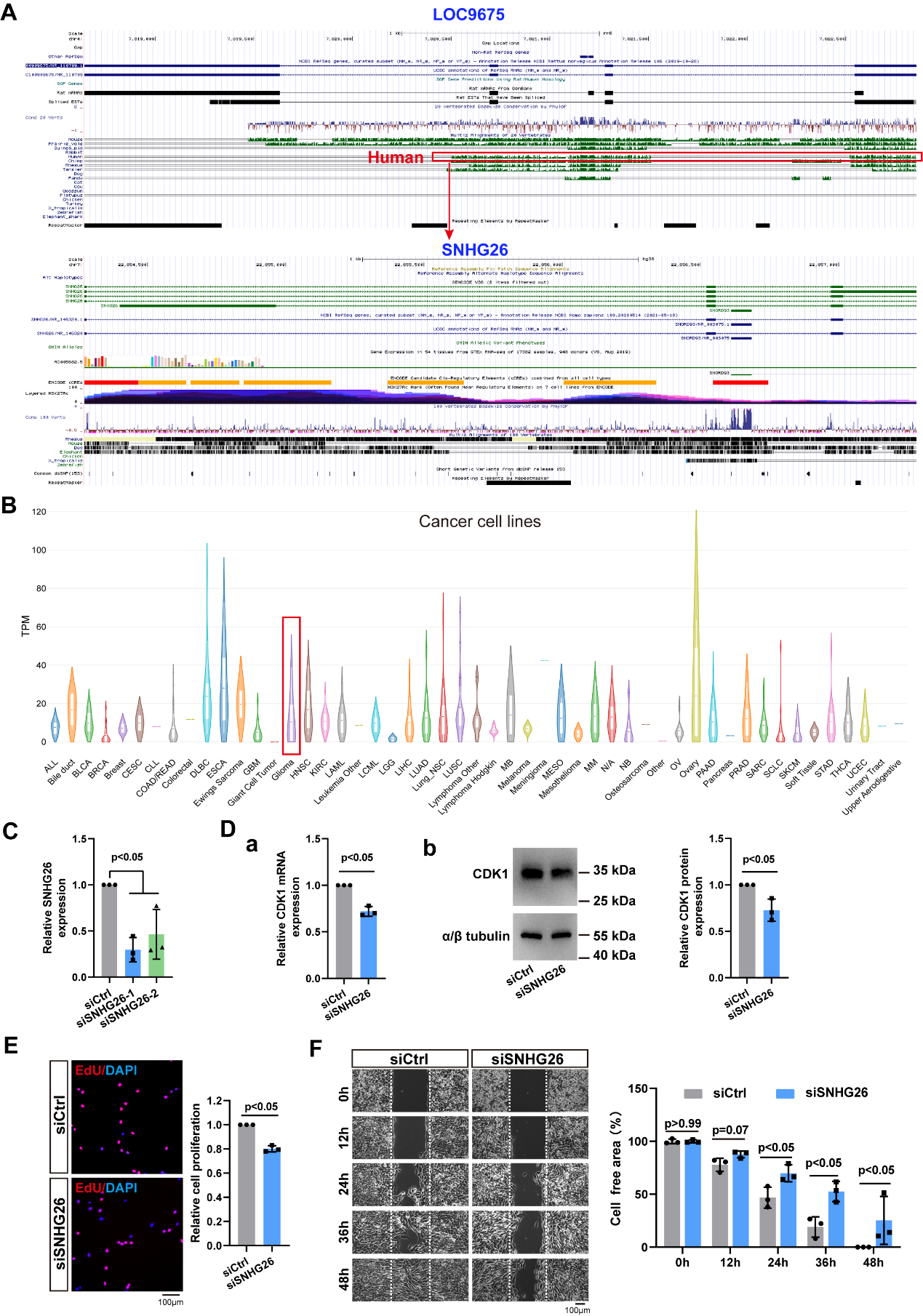


**Supplementary Figure S5. Knockdown of SNHG26 significantly reduced cell proliferation, migration, and the expression of CDK1 in U251**

A, UCSC browser image of lncRNA LOC9675 and SNHG26.

B, The expression of SNHG26 in cancer cell lines.

C, Graph showing knockdown efficiency of SNHG26 small interfering RNA (siRNA). After siRNA-1 treatment for 24 h, relative level of LOC9675 expression decreased by 70.3%, whereas siRNA-2 treatment decreased by 53.7%. The data are shown as the mean ± SD. Data were analyzed using one-way ANOVA followed by Tukey’s post hoc test (*p*<0.05). Unpaired Student's t test was used to compare two groups (vs. siCtrl, n = 3). The *p* values are shown on the panel.

D, Panel a: qPCR results for CDK1 after siSNHG26 treatment for 48 h. Panel b, western blotting results for CDK1 after siSNHG26 treatment for 48 h. The data are shown as the mean ± SD. Data were analyzed using unpaired Student’s t test (vs. siCtrl, n = 3). The *p* values are shown on the panel.

E, 5-Ethynyl-2′-deoxyuridine (EdU) results after SNHG26 siRNA treatment. Left panel: Representative EdU images. Right panel: Statistical analysis. The data are shown as the mean ± SD. Data were analyzed using unpaired Student’s t test (vs. siCtrl, n = 3). The *p* values are shown on the panel.

F, Ibidi chamber assay results after SNHG26 siRNA treatment. Left panel, representative images. Right panel, statistical analysis. The data are shown as the mean ± SD. Data were analyzed using two-way ANOVA followed by Bonferroni's post hoc test (*p*<0.05). Unpaired Student's t test was used to compare two groups (vs. siCtrl, n = 3). The *p* values are shown on the panel.


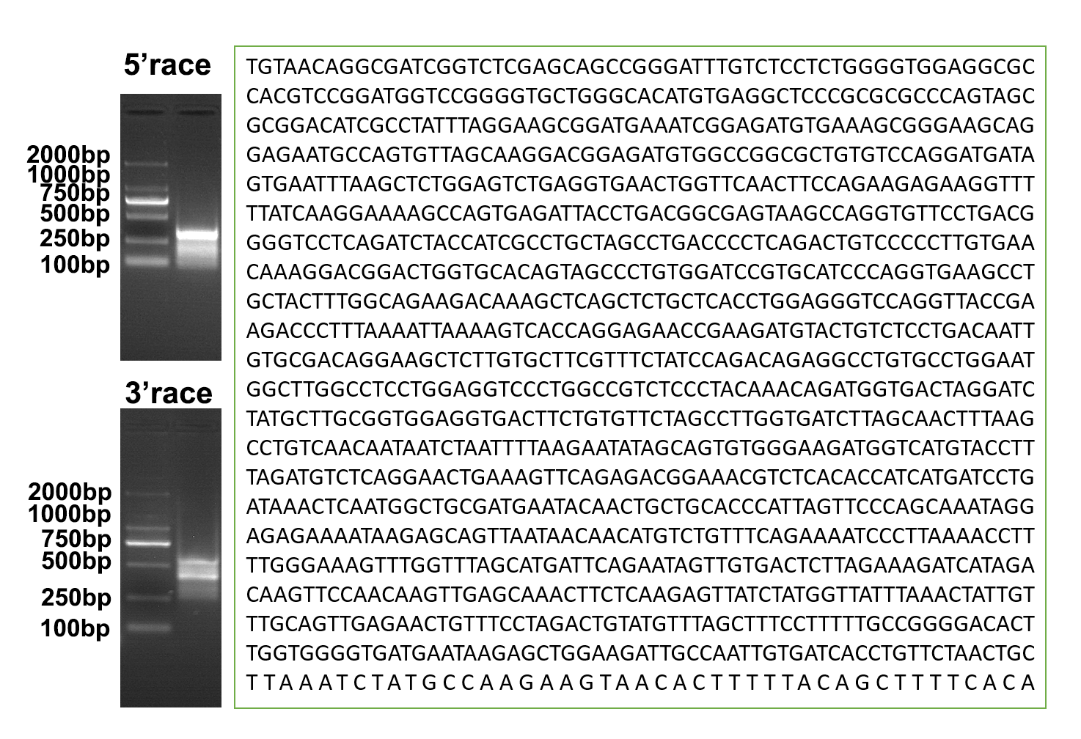


**Supplementary Figure S6. The full length of LOC9675S by RACE**


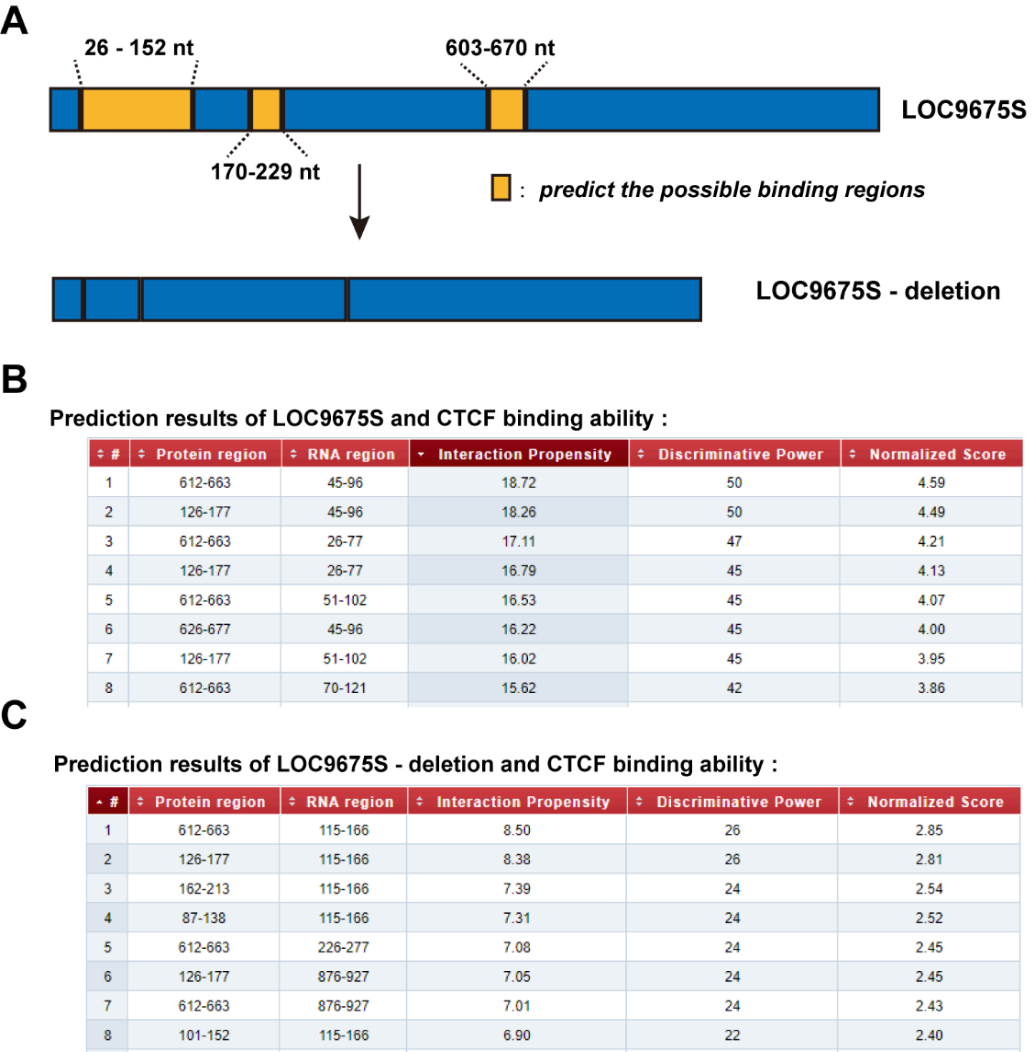


**Supplementary Figure S7. The constructs of LOC9675S mutant plasmid**

A, Schematic diagram of LOC9675S mutant plasmid synthesis.

B, Prediction results for LOC9675S and CTCF binding ability using the catRAPID website.

C, Prediction results of LOC9675S-deletion and CTCF binding ability using the catRAPID website.

**Raw data for Western blot analysis**


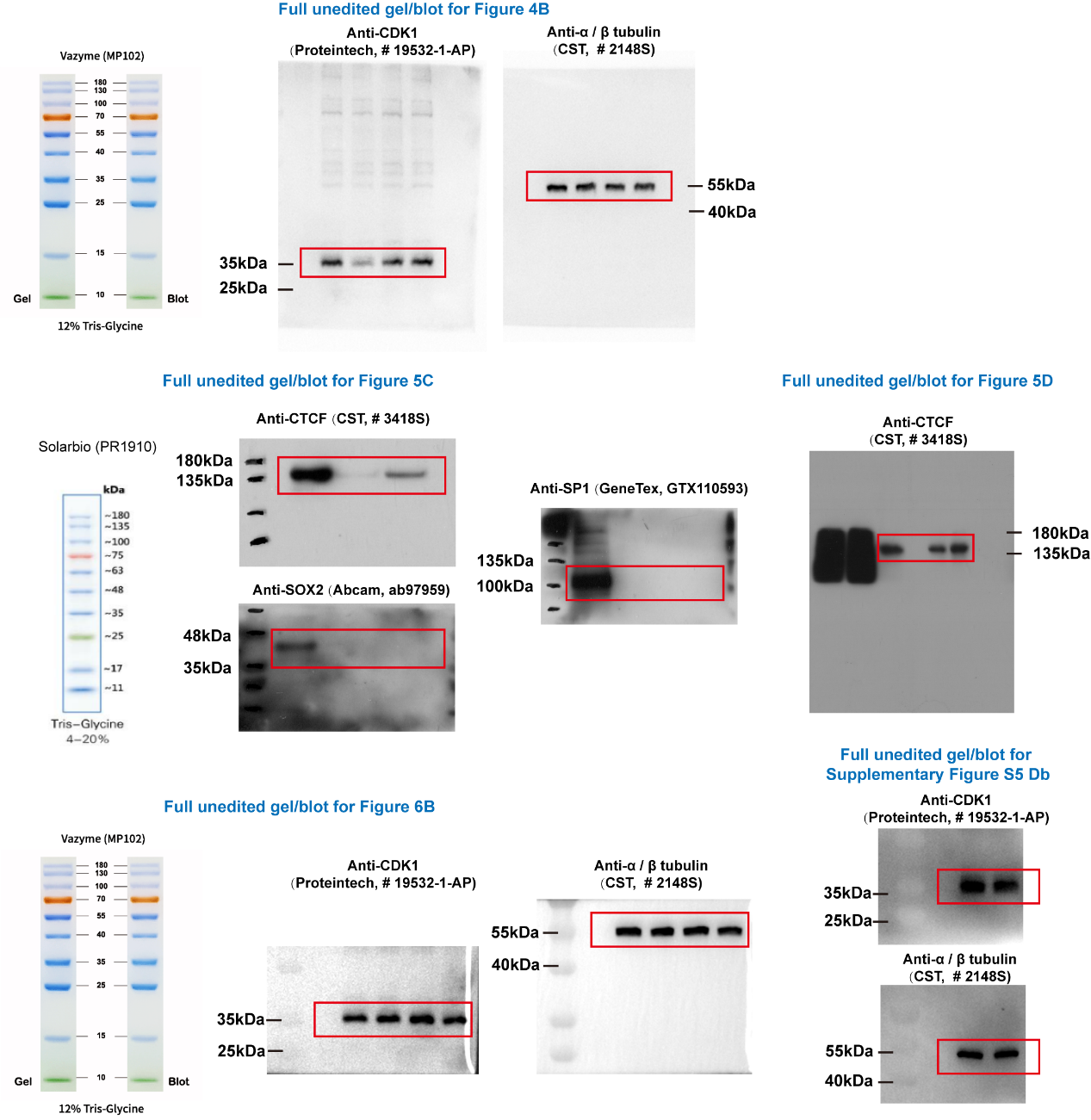

Supplement: Supporting Figures S1–S7 [file mmc1.docx]
